# UMI-count modeling and differential expression analysis for single-cell RNA sequencing

**DOI:** 10.1186/s13059-018-1438-9

**Published:** 2018-05-31

**Authors:** Wenan Chen, Yan Li, John Easton, David Finkelstein, Gang Wu, Xiang Chen

**Affiliations:** 1 Department of Computational Biology, St. Jude Children’s Research Hospital, 262 Danny Thomas Pl, Memphis, TN 38105 USA; 2 Division of Biostatistics, School of Public Health, University of Minnesota Twin Cities, Mayo Building, Minneapolis, MN 55455 USA

**Keywords:** Unique molecular identifier, Negative binomial, Differential expression analysis

## Abstract

Read counting and unique molecular identifier (UMI) counting are the principal gene expression quantification schemes used in single-cell RNA-sequencing (scRNA-seq) analysis. By using multiple scRNA-seq datasets, we reveal distinct distribution differences between these schemes and conclude that the negative binomial model is a good approximation for UMI counts, even in heterogeneous populations. We further propose a novel differential expression analysis algorithm based on a negative binomial model with independent dispersions in each group (NBID). Our results show that this properly controls the FDR and achieves better power for UMI counts when compared to other recently developed packages for scRNA-seq analysis.

**Electronic supplementary material:**

The online version of this article (10.1186/s13059-018-1438-9) contains supplementary material, which is available to authorized users.

## Background

Single-cell RNA-sequencing (scRNA-seq) technology provides transcriptome profiles of individual cells, enabling the dissection of the heterogeneity of different cell populations and tissues [1]. Although scRNA-seq protocols share common principles of single-cell isolation, cell lysis, transcript capture, complementary DNA (cDNA) conversion and amplification, library preparation, and sequencing, the methodologies differ. Multiple methods for transcript quantification with differing levels of accuracy and sensitivity have been employed in scRNA-seq analysis [2]. However, the paucity of starting material for reverse transcription remains an inherent limitation of scRNA-seq protocols and contributes to the relatively low rate at which messenger RNA (mRNA) molecules in individual cells are converted to cDNA molecules that can be captured and sequenced [3, 4]. Coupled with the stochastic nature of gene expression, scRNA-seq protocols generally produce single-cell transcriptome measurements with low signal-to-noise ratios, exemplified by the high abundance of zeroes in the expression matrix and so-called dropout events. In this context, dropout refers to a special type of missing value whereby the expression of a gene is detected at a moderate or high level in a subset of cells but is not detected in other cells [5].

Read counts and transcript counts are two categories of quantification schemes commonly employed in scRNA-seq. Although the read count-based scheme is similar to the common approaches used for bulk RNA-seq, the miniscule quantity of transcripts captured from a single cell requires cDNA amplification for library construction; this inevitably results in large amplification bias [6]. To mitigate this bias, several recent scRNA-seq protocols have employed an additional step in which individual transcripts are barcoded with unique molecular identifiers (UMIs) before amplification, resulting in a more accurate quantification of the transcript count [7, 8].

Although the fast-evolving experimental protocols for scRNA-seq have given rise to numerous studies employing scRNA-seq techniques, statistical characterizations of scRNA-seq data continue to lag. Most published studies have focused primarily on either read counts [5, 9, 10] or UMI counts [3, 7]. Although a few studies that used both read-count and UMI-count schemes have suggested that employing UMIs in expression measurement globally reduces the technical noises and that the data generally fit into simpler statistical models compared to read counts [3, 11–13], a popular perspective held by the field is that dropout events result in more zeroes than expected in scRNA-seq data and these events need to be explicitly modeled using zero inflated/bimodality models [5, 10, 14–17]. This study investigated the necessity and effectiveness of zero-inflated models in modeling the UMI-count distribution among cells by directly comparing the statistical modeling of UMI counts and read counts.

A closely related application of scRNA-seq count modeling is single-cell differential expression (DE) analysis. Several software packages have been developed specifically for scRNA-seq DE analysis, such as SCDE [5], MAST [9], ROTS [18], Monocle2 [11], and Seurat [19]. However, there have been no systematic evaluations of these methods with respect to UMI count-based scRNA-seq data.

In this study, we first conducted a comprehensive analysis of the modeling UMI counts and read counts in scRNA-seq data. Based on that analysis, we proposed a method using the Negative Binomial model with Independent Dispersions (NBID) and compared its false discovery rate (FDR) control and power to those of other commonly used methods. We also illustrate a practical application of NBID in biomarker identification after unsupervised clustering of scRNA-seq data.

## Results

### Model comparison for UMI counts and read counts

**Fig. 1:**
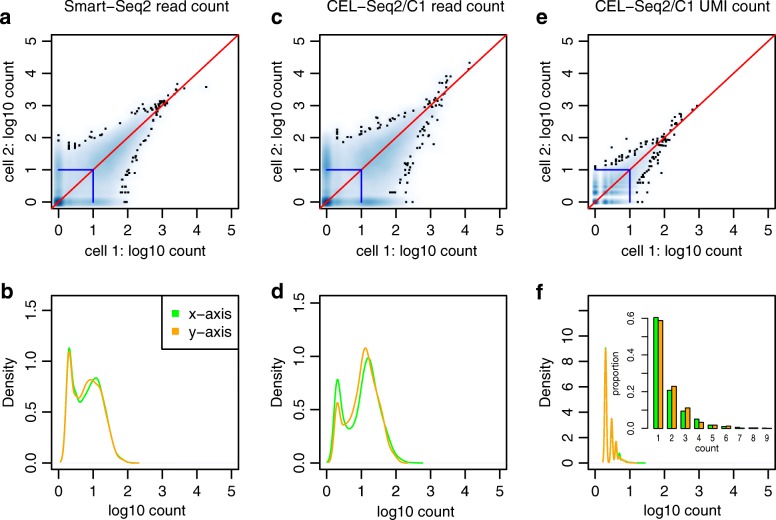
*Scatter plots* of two cells with similar read counts or UMI counts. **a**, **b** Read counts for Smart−Seq2. **c**, **d** Read counts for CEL − Seq2/C1. **e**, **f** UMI counts for CEL − Seq2/C1. **a**, **c**, **e** The *scatter plot* with color-coded density, the highest density at the origin. The *left* and *middle panels*, which are based on the read counts, show very different patterns from the *right panel*, which is based on the UMI counts. **b**, **d**, **f** The density plot along the x- and y-axes of (**a**), (**c**), and (**e**), excluding the origin. For all plots, we kept the genes that were detected in at least five cells among all cells

We used a unique dataset produced by Ziegenhain et al. [12] to determine the difference between read counts and UMI counts. A homogeneous population of mouse embryonic stem cells was derived by two inhibitors/leukemia inhibitory factors and used to evaluate six different scRNA-seq protocols, including four UMI count-based protocols and two read count-based protocols. Furthermore, the read counts before conversion to UMIs were also evaluated for the four UMI based protocols, which provided an excellent opportunity to examine the differences between the UMI count and read count for the same data [12]. We first examined scatter plots for cell pairs with similar total read/UMI counts. Figure 1 shows the representative pattern for two cells. This general pattern holds for most cell pairs in the dataset. A large density mass was focused on at (0, 0) (the origin point) in all three results (Fig. 1a, c, and e), which was consistent with the notion that transcripts for most genes were not captured by either cell in scRNA-seq protocols (supported by abundance of zeros in the scRNA-seq data) [10]. Read-count measurements produced results (Fig. 1a–d) similar to those of Kharchenko et al. [5], which were used to illustrate dropout events. The overall UMI-count measurements (Fig. 1e and f) showed less divergence when compared to their read-count counterparts in the same cell pair (Fig. 1c and d). Specifically, quantifications for genes with dropout events (i.e. when transcripts/reads were captured in one cell in the pair but not the other) showed a distinct bi-modal pattern in the read counts (Fig. 1b and d) but a unimodal distribution in the UMI counts (Fig. 1f). Consequently, it is prudent to model gene expression by using a zero-inflated model (i.e. a zero-inflated negative binomial [ZINB] model [5] or hurdle model [9]) for the read counts: one component for the zero counts and the other component for the non-zero counts. However, the fast attenuation of density along the axes suggests that a unimodal distribution (e.g. a Poisson or negative binomial [NB] distribution) may be sufficient for UMI counts.

To further capture the quantitative difference between read counts and UMI counts, we modeled them with different distributions. We employed a backward selection strategy on three candidate models that are commonly used in scRNA-seq studies [3, 5, 7]: a Poisson model with one parameter defining both the mean and the variance; a NB model with two parameters defining the mean and variance; and a ZINB model with three parameters, of which two were the same as those in the NB model. The additional parameter in the ZINB model defines the probability of a count being zero or being distributed as an NB distribution. These three models are nested with increasing complexity, i.e. the Poisson model is a special case of the NB model and the NB model is a special case of the ZINB model. Our goal was to decide on the proper complexity for fitting the scRNA-seq data. We started by testing whether the ZINB model was significantly better than the NB model for modeling the counts. Among those genes that did not reject the NB model, we further tested whether that model was significantly better than the Poisson model. The model selection results are summarized in Table 1 (see also Additional file 1: Figure S1). We analyzed both the UMI counts and read counts (before UMI conversion) form four UMI-based protocols. Although no genes measured in UMI counts preferred the ZINB model over the NB model at an FDR level of 0.05, the results for CEL-Seq2/C1 and MARS-Seq showed a significant percentage of genes (9.4–34.5%) rejecting the NB model in favor of the ZINB model when measured in read counts. Moreover, for UMI counts, a large proportion of genes (39.4–84.0%) selected the simple Poisson distribution. By contrast, read-count measurements resulted in a sharp drop in the proportions of Poisson models (2.4–9.5%, *p* = 0.0078, the Wilcoxon signed rank test) across all platforms evaluated. Read-count only protocols (Smart-Seq and Smart-Seq2) show comparable patterns to the read counts from UMI protocols. Overall, our analysis implies that while ZINB is necessary for a significant fraction of read counts, it is not needed for UMI counts.

**Table 1:** Number of genes with selected models for different protocols from Ziegenhain et al. [12]

					NB vs ZINB		Poisson vs NB	
Protocol	Cells used (n)	Genes detected (n)	Genes tested (n)	Genes converged (n)	ZINB	NB	Poisson	Poisson (%)
UMI count								
CEL-Seq2/C1(A)	34	16,690	11,345	11,345	0	2968	8377	73.84
CEL-Seq2/C1(B)	37	17,229	12,190	12,189	0	3794	8395	68.87
Drop-Seq(A)	42	16,579	10,702	10,702	0	4277	6425	60.04
Drop-Seq(B)	34	15,469	9288	9288	0	3659	5629	60.61
MARS-Seq(A)	29	14,551	8266	8266	0	4592	3674	44.45
MARS-Seq(B)	36	15,406	9644	9644	0	5848	3796	39.36
SCRB-Seq(A)	39	16,411	12,955	12,955	0	1214	11,741	90.63
SCRB-Seq(B)	45	16,944	13,212	13,212	0	2115	11,097	83.99
CEL-Seq2/C1(A)	34	16,690	11,345	10,679	3679	6385	615	5.76
CEL-Seq2/C1(B)	37	17,229	12,190	12,155	1174	10,443	538	4.43
Drop-Seq(A)	42	16,579	10,702	10,690	121	9601	968	9.06
Drop-Seq(B)	34	15,469	9288	9278	91	8329	858	9.25
MARS-Seq(A)	29	14,551	8266	8132	761	7161	210	2.58
MARS-Seq(B)	36	15,406	9644	9531	1333	7974	224	2.35
SCRB-Seq(A)	39	16,411	12,955	12,954	0	11,814	1140	8.80
SCRB-Seq(B)	45	16,944	13,212	13,212	0	11,964	1248	9.45
Read count								
Smart-Seq2(A)	80	21,076	15,294	15,098	7905	5795	1398	9.26
Smart-Seq2(B)	77	20,861	15,224	15,152	6456	7244	1452	9.58
Smart-Seq/C1(A)	69	19,699	13,518	13,513	16	12,761	736	5.45
Smart-Seq/C1(B)	61	19,100	12,949	12,947	0	11,888	1059	8.18

### Negative binomial model for UMI counts

A model selection strategy always selects a “best” model among the specified candidates even though the chosen model may fit the underlying data poorly. Therefore, we evaluated the goodness of fit for these selected models. Because a Poisson model can be modeled as a special scenario of the NB model, we began by measuring the goodness of fit of the NB model for various datasets reported by Ziegenhain et al. [12] (Table 2). At an FDR level of 0.05, only 0.1% (range = 0–0.4%) of converged genes rejected the NB model for UMI counts. This percentage was significantly increased to 14.2% (range = 1.1–35.3%, *p* = 0.0078, the Wilcoxon signed rank test) for read counts from the same datasets, indicating that a high-level noise was introduced by cDNA amplification. We further examined the proportion of genes that could be modeled by a Poisson model. As expected, the percentage of genes with an adequate Poisson fit (FDR > 0.05) dropped sharply from 80.2% (range = 65.7–95.1%) for UMI counts to 2.6% (range = 1.0–4.1%, *p* = 0.0078, the Wilcoxon signed rank test) for read counts measured in the same datasets. The goodness of fit of both the Poisson and NB models supports the conclusion that UMI counts can be modeled by simpler models when compared to read counts.

**Table 2:** Goodness of fit test for the Poisson and NB models for different protocols from Ziegenhain et al. [12]

Protocol	Cells Used (n)	Genes tested (n)	Genes reject Poisson (n)	Genes reject NB (n)	Accept Poisson (%)	Reject NB (%)
UMI count						
CEL-Seq2/C1(A)	30	3357	660	1	80.34	0.03
CEL-Seq2/C1(B)	33	5601	1082	3	80.68	0.05
Drop-Seq(A)	37	2311	548	2	76.29	0.09
Drop-Seq(B)	30	1690	414	0	75.50	0.00
MARS-Seq(A)	26	1162	317	2	72.72	0.17
MARS-Seq(B)	32	2184	750	8	65.66	0.37
SCRB-Seq(A)	35	4218	214	1	94.93	0.02
SCRB-Seq(B)	40	4360	213	0	95.11	0.00
Read count before converting to UMI						
CEL-Seq2/C1(A)	30	6012	5954	622	0.96	10.35
CEL-Seq2/C1(B)	33	7993	7897	90	1.20	1.13
Drop-Seq(A)	37	4574	4386	430	4.11	9.40
Drop-Seq(B)	30	2867	2781	512	3.00	17.86
MARS-Seq(A)	26	2830	2743	152	3.07	5.37
MARS-Seq(B)	32	4248	4168	265	1.88	6.24
SCRB-Seq(A)	35	7392	7194	2065	2.68	27.94
SCRB-Seq(B)	40	7112	6855	2507	3.61	35.25
Read count						
Smart-Seq2(A)	72	10,880	10,692	2696	1.73	24.78
Smart-Seq2(B)	69	10,684	10,469	1790	2.01	16.75
Smart-Seq/C1(A)	62	9342	9249	87	1.00	0.93
Smart-Seq/C1(B)	55	7990	7893	75	1.21	0.94

A model is rejected if FDR < 0.05 among all genes tested; otherwise it is accepted

*NB* negative binomial

### Modeling and goodness of fit for UMI counts in large scale scRNA-seq datasets

Although the datasets of Ziegenhain et al. [12] provided an unparalleled opportunity to evaluate the difference between read counts and UMI counts, the number of cells captured was relatively small (range = 29–80). We extended our analysis to additional datasets generated by different platforms [7, 20–23] to evaluate whether the same pattern generally held for other datasets. Despite technical differences among protocols and heterogeneity within cell populations, overall, the model selection and goodness-of-fit analysis for these datasets supported our conclusion that UMI counts can be modeled by simpler models when compared to read counts (Additional file 2: Tables S1A and S1B).

Since 2016, several Drop-seq UMI based platforms have appeared with the capability to process thousands of cells in a single experiment [2, 8]. Consequently, we studied whether the same pattern held for such large-scale datasets. We applied the described model-selection strategy and goodness-of-fit test to the following datasets: (1) CD4+ naïve T cells (9850 cells); and (2) CD4+ memory T cells (9578 cells), both of which were generated on the GemCode platform (10× Genomics, Pleasanton, CA, USA) [8], and 3) Rh41 cells, a human *PAX3-FOXO1* positive alveolar rhabdomyosarcoma (ARMS) cell line (6875 cells) prepared in-house on the Chromium platform (10× Genomics). Rh41 cells contained two distinct subpopulations based on unsupervised clustering analysis (Additional file 1: Figure S2) and were included to evaluate the effects of strong heterogeneity on model selection and fitting (Table 3). Although few genes (4–7, 0.04–0.06%) preferred the ZINB model in the relatively homogeneous T-cell populations, the percentage of genes selecting the ZINB model in Rh41 cells was slightly elevated, albeit still low (39 genes, 0.21%). The expression of these genes differed significantly between the two clusters (FDR < 0.05, the Wilcoxon rank sum test; see also Additional file 2: Table S2), suggesting that the fraction of genes preferring the ZINB model correlates with the level of heterogeneity.

**Table 3:** Number of genes with selected models for large-scale datasets on the GemCode and Chromium platforms

					NB vs ZINB	Poisson vs NB		
Data	Cells used (n)	Genes detected (n)	Genes tested (n)	Genes converged (n)	ZINB	NB	Poisson	Poisson (%)
Naive T cells (Gemcode)	9850	32,738	11,978	11,977	7	5336	6634	55.39
Memory T cells (Gemcode)	9578	32,738	12,569	12,567	4	6336	6227	49.55
Rh41 (Chromium)	6875	33,416	18,435	18,435	39	9387	9009	48.87

**Fig. 2:**
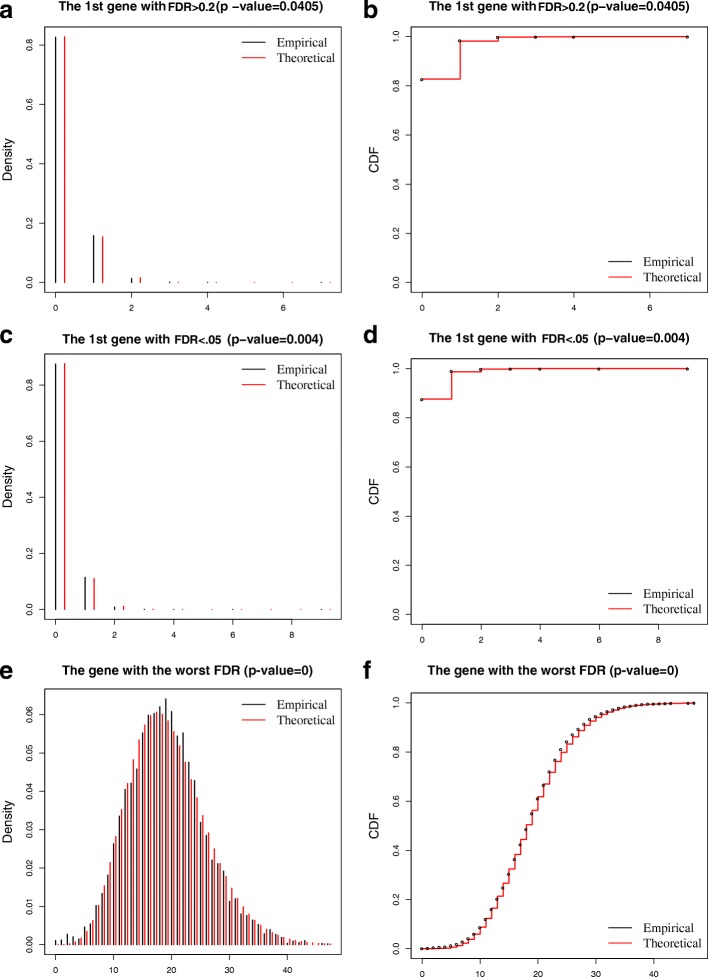
Goodness of fit using the negative binomial distribution on the naïve T-cell data (Tn). **a** The empirical and theoretical probability mass function (pmf) for the first gene with FDR > 0.2. **b** The empirical and theoretical cumulative distribution function (cdf) for the first gene with FDR > 0.2. **c, d** The same pmf and cdf plots for the first gene with FDR < 0.05. **e, f** The same pmf and cdf plots for the gene with the worst FDR

Compared to the datasets of Ziegenhain et al. [12], the T and Rh41 datasets displayed a lack of statistical fit for simpler models (Table 4). Specifically, the genes modeled by the Poisson model dropped to 61.1% (range = 51.9–67.6%) and the percentage of genes that rejected the NB model increased to 5.3% (range = 3.4–8.4%). In addition to elevated heterogeneity in the Rh41 cells, the sample size of these datasets (range = 6875–9850) also played an important role in the increased lack of model fitness. It has been documented that very large samples invariably produce statistically significant lack of fit, even though the departure from the specified distributions may be very small and unimportant [24]. Therefore, we compared the empirical probability mass function (pmf) and the cumulative distribution function (cdf) with the fitted negative binomial model to evaluate visually the difference between them for genes rejecting the NB model (Fig. 2, Additional file 1: Figures S3 and S4). Even though these genes rejected the NB model at an FDR level of 0.05, the fitted pmf and cdf curves were good approximations of their empirical counterparts. Importantly, among the 23 to 282 genes that rejected the NB model, only few (3–17) were adequately approximated by the ZINB model (Additional file 2: Table S3). Therefore, we conclude that the NB model is a good approximation model for UMI counts, even for large-scale scRNA-seq data with evidence of heterogeneity.

**Table 4:** Goodness of fit test for the Poisson and NB models for large-scale datasets on the GemCode and Chromium platforms

Data	Cells used (n)	Genes tested (n)	Genes reject Poisson (n)	Genes reject NB (n)	Accept Poisson (%)	Reject NB (%)
Naive T cells (Gemcode)	7332	776	403	65	48.07	8.38
Memory T cells (Gemcode)	8622	836	533	28	36.24	3.35
Rh41 (Chromium)	6187	6853	4630	295	32.44	4.30

A model is rejected if FDR < 0.05 among all genes tested; otherwise it is accepted

*NB* negative binomial

### scRNA-seq differential expression analysis

A direct consequence of properly modeling scRNA-seq counts is the power to accurately conduct differential expression analyses. Based on the knowledge derived from UMI-count modeling, we proposed a NB-based algorithm for differential expression analysis of large-scale UMI-based scRNA-seq data. We extended the general NB-based models by allowing independent dispersion parameters in each biological condition, resulting in the NBID method. This approach is analogous to the *t*-test, which allows different variances between groups when testing the equivalence of means. The rationale stems from the apparent variations in dispersion even at the same average expression level [3, 7]. Because the number of cells in each condition is generally sufficient in large-scale datasets, we derive separate dispersion estimates for each condition; these are used in the subsequent NB-based test against the null hypothesis that different conditions have the same average expression. We compared the proposed method with other commonly used methods (Additional file 2: Table S4): Monocle2 [11]; SCDE [5]; ROTS [18]; MAST [9]; and Seurat [19]. Although both SCDE and MAST were developed for read counts, their authors claim that they can be applied to UMI data. To handle the apparent zero inflation, SCDE employs a mixture of a NB model and a Poisson model, while MAST uses a hurdle model with the non-zero component modeled with a Gaussian distribution. Monocle2 [11] and Seurat [19] provide NB-based differential expression analysis (among other models) for UMI counts. We also included ROTS based on a recent comparison of scRNA-seq differential expression analysis [18]. Recently, several scRNA-seq-tailored normalization schemes have been proposed [16]. We evaluate their contributions by integrating NBID with scran, a state-of-the-art generic normalization method [25], by using the cell-specific size factor estimated by scran in NBID (NBID_scran).

### FDR and power comparison for differential expression analysis of UMI-based scRNA-seq data

We first evaluated the FDR control for all methods by using simulated data. Instead of generating artificial datasets from a theoretical distribution, we simulated groups of cells with differentially expressed genes from publicly available datasets collected from different protocols (data from memory T cells obtained by GemCode [8], from whole-intestinal organoids obtained by CEL-Seq [20], and from heterogeneous dendritic cells obtained by MARS-Seq [21]). We began with randomly generating two distinct groups of cells by swapping the UMI counts for two sets of genes in the second group. Here, the first group represented cells collected under a reference condition and the second group contained cells under the testing condition with simulated differential expressions. The two equal-sized sets of genes had different average expression levels in the full dataset before swapping. This strategy generated artificially separated groups of cells while retaining specific characteristics of the scRNA-seq counts for each cell. The distribution of the total number of UMIs captured in a cell is an important characteristic for UMI-based scRNA-seq experiments. Although biological differences (such as the physical cell size, proliferation status, and cell-cycle stages) may affect the absolute number of transcripts in the cells, technical (non-biological) variations, such as the cell-to-cell variations in the conversion factor between transcripts and captured UMIs and variations in the sequencing depth, have substantial influence on the number of UMIs captured for each cell. Moreover, the effects of total UMI variations are disproportionally biased towards the gene with lower expression [17, 26] and the disparity in the number of UMIs is further exacerbated in scenarios in which two groups of cells being compared are captured and sequenced separately. To evaluate the robustness of performance against the commonly observed difference in the total UMIs captured per cell, we simulated three scenarios in terms of expected group difference in the total UMIs captured by sub-sampling UMIs in the second group of cells: no difference (100% UMIs retained); mild difference (80–90% UMIs retained); and intermediate difference (50–60% UMIs retained). Because NBID assumes a sufficiently large number of cells in each group, we evaluate its robustness in common scenarios for scRNA-Seq experiments with different number of cells (60, 300, or 1000 cells, approximating samples collected from 96-well plates, 384-well plates, and by droplet methods, respectively).

In extensive simulations of 300 or 1000 cells (two groups combined) with different simulated fold change from multiple datasets obtained by different protocols, Monocle2, SCDE, and Seurat consistently inflated the FDR and the number of false positives increased with the level of expected UMI difference between groups (Table 5, Additional file 2: Tables S5–S8). Similar to the comparisons result derived from read counts [27], SCDE generally detected fewer DE genes compared to other methods in UMI-count scRNA-seq data. However, it often produced relatively high number of false positives, which resulted in severely inflated FDRs in the simulations. Due to the severely inflated FDRs (with or without expected group difference in the total UMI counts) in many scenarios, both SCDE and Seurat were excluded from subsequent analyses. ROTS controlled FDR without UMI difference, but severely inflated the FDR in scenarios with expected group differences in UMIs. While MAST controlled the FDR without and with mild group difference in UMIs, it also shows inflated the FDR with intermediate differences in total UMIs. NBID and NBID_scran were the only methods to achieve proper FDR control under all three scenarios (Table 5).

**Table 5:** FDRs of evaluated methods

		1^a^		0.8–0.9^b^			0.5–0.6^c^		
Method	FDR	False (n)	DE (n)	FDR	False (n)	DE (n)	FDR	False (n)	DE (n)
Monocle2	**0.069**	5.9	83.9	**0.089**	7	79.1	**0.276**	22.1	79
SCDE	**0.299**	2.6	8.3	**0.34**	3.7	9.5	**0.848**	123.5	145.2
MAST	0.001	0	29.5	0.003	0.1	28.2	**0.193**	3.4	19.5
ROTS	0.045	4.4	97.5	**0.497**	71.6	145.9	**0.835**	272.4	323.9
Seurat_ttest	**0.244**	31.5	128.3	**0.441**	69.6	156.1	**0.927**	653.6	704.5
Seurat_bimod	**0.154**	17.6	112.5	**0.655**	172	258.7	**0.928**	924.5	996.3
Seurat_tobit	**0.248**	32.2	129	**0.45**	72	158.5	**0.873**	351.7	402.8
Seurat_poisson	**0.208**	25.7	122.6	**0.188**	20.1	106.1	**0.573**	67.4	116.2
Seurat_negbinom	**0.197**	23.9	120.7	**0.164**	16.9	102.4	**0.5**	47.7	93.8
NBID_scran	0.038	3.4	86.9	0.035	2.8	80.1	0.039	2.5	62.4
NBID	0.033	2.8	85.7	0.032	2.7	81.4	0.03	1.8	58.9

^a^No sub-sampling

^b^The sub-sampling ratio in Group 2 was 0.8–0.9

^c^The sub-sampling ratio in Group 2 was 0.5–0.6.

Bold values indicate FDR > 0.05. Bold and underlined values indicate FDR > 0.1. The nominal FDR was 0.05. Simulation based on the Memory T-cell data [8], 500 cells in each group, results are averaged over 96 replicates (see Additional file 2: Tables S5–S9 for results for other simulation scenarios). NBID_scran used the size factor computed by scran as the offset instead of the total UMI counts

In the simulation of 60 cells combined, all the methods except for MAST yielded various degrees of FDR inflation (Additional file 2: Table S9), indicating that > 60 cells are needed for a robust DE analysis in scRNA-seq. Nevertheless, when we focused on genes with high expression (with TPM ≥ 50 in at least one group, similar to the threshold employed in reference [28]), NBID and NBID_scran approached the desired FDR control in all three scenarios.

**Fig. 3:**
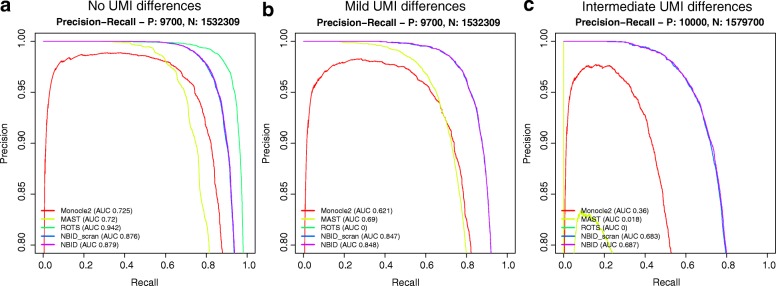
*Precision-recall curves* for selected methods. **a** The precision-recall curve without UMI differences between two groups. **b** The precision-recall curve with mild UMI differences between two groups. **c** The precision-recall curve with intermediate UMI differences between two groups. For each scenario, some methods failed to run on few replicates but at least 97 replicates were used to calculate the precision and recall rate. P true DE genes, N true non-DE genes

We used precision-recall curves to evaluate the power of the methods (Fig. 3 and Additional file 1: Figures S5–S9). Measured by the area under the curve (AUC), NBID and NBID_scran robustly outperformed other methods in different simulation scenarios. Although ROTS had a slight edge without group difference in UMIs, it was highly sensitive to the group difference: even a mild difference dropped the AUC to 0 in simulations with 1000 cells. NBID and NBID_scran achieved similar results, suggesting that the total UMI count is a good estimator for the cell-specific size factor in the simulations.

### Differential expression analysis of naive T cells and memory T cells

**Fig. 4:**
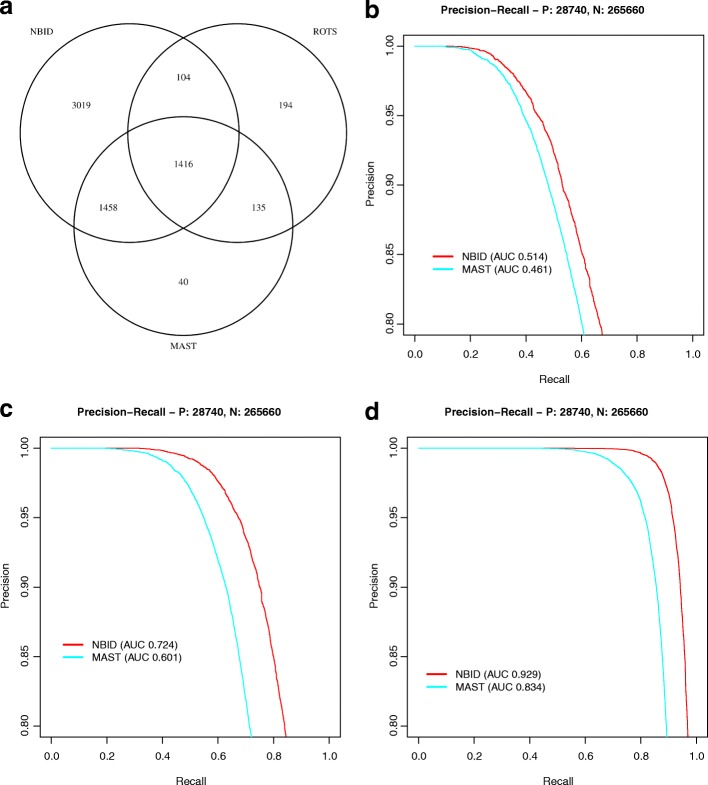
Comparison of detected genes in naïve T cells and memory T cells. **a** The *Venn diagram* of DE genes detected by NBID, ROTS, and MAST. **b** The *precision-recall curves* obtained using a subset of 1000 cells of each cell type. For the power calculation, we chose the DE genes detected in both NBID and MAST as the true DE genes and the genes not detected as DE genes in either NBID or MAST as the true non-DE genes. **c**, **d** The same as (**b**) except using subsets of 2000 and 5000 cells, respectively

We evaluated three algorithms (MAST, ROTS, and NBID) for their ability to identify DE genes in naïve T cells and memory T cells (Fig. 4 and Additional file 2: Tables S10 and S11). NBID detected more DE genes than did MAST or ROTS (Fig. 4a), consistent with the simulation results showing better power with NBID. Because the true DE genes in the groups were unknown, we compared the inferred DE genes against a published list of DE genes for naïve T cells and memory T cells (Table 1 in reference [29]). Since both T-cell datasets were derived from the CD4+ population [8], CD8+ specific genes were ignored. Of the 37 true positives, NBID, MAST, and ROTS recovered 34 (92%), 30 (81%), and 24 (65%), respectively. The three genes missed by NBID (*LY96*, *STAM*, and *TOX*) had very low expression in the dataset (average UMI count from the large group: 0.002, 0.007, and 0.007, and TPM: 2.7, 9.4 and 10.7, with an average of approximately 850 UMIs being captured per cell), leading to insufficient detection power for DE genes. Consequently, none of the evaluated algorithms classified the three genes as DE genes. Additional file 1: Figure S10 shows density plots of selected genes.

We carried out additional in-silico validation of predicted DE genes by NBID and MAST. We assumed that the DE genes detected by both algorithms were true positive and that genes not detected as DE genes by either algorithm were true negatives. We then randomly sampled subsets of cells from each population (1000, 2000, or 5000 cells) and compared the recovery of these genes in ten subsampled replicates. NBID outperformed MAST, having a higher AUC in all three settings (Fig. 4b–d).

In this real-data analysis, NBID_scran again achieved similar results as NBID. Specifically, 5728 DE genes were detected by both methods, accounting for 95.5% and 94.3% of all DE genes by NBID (5997) and NBID_scran (6076), respectively. Together with additional evidences from simulation studies, we conclude that the default normalization scheme employed by NBID generally achieved comparable performance with scran, a state-of-the-art normalization scheme.

### Differential expression analysis for biomarker identifications

**Fig. 5:**
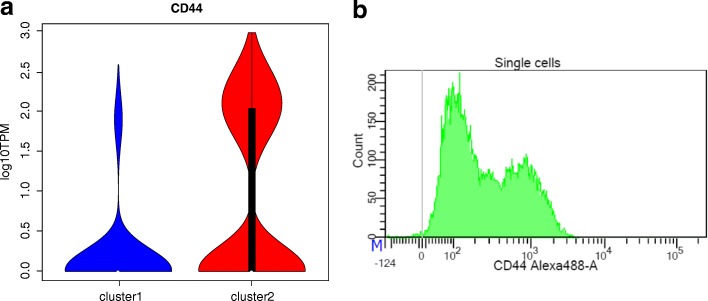
Differential expression of CD44 in two clusters in the Rh41 cell line. **a** *Violin plot* of the gene expression among cells in the two clusters, the TPM is in log10 scale after adding a small value 1. **b** The CD44 count distribution when using CD44 to sort single cells, indicating two clusters of cells with different levels of CD44 expression

scRNA-seq has been widely used to reveal the subpopulation structure in heterogeneous cell populations through unsupervised clustering approaches [1]. Differential expression analysis of identified cell subpopulations can further characterize their functional differences and identify potential biomarkers for experimental validation and subpopulation separation. Consequently, we applied NBID and MAST to detect DE genes in the two subpopulations inferred to be present in Rh41 cells. Among the expressed genes (with TPM ≥ 3 in at least one group [30]), NBID and MAST revealed 1019 and 448 DE genes between the two clusters with a fold change > 2 between the two clusters (Additional file 2: Tables S12, S13), respectively. We ranked the potential of DE genes to be robust biomarkers based on the test FDR values, their relative fold changes, and their overall expression levels. The *CD44* gene, which encodes a commonly used cell surface marker, appeared at the top of the list (Fig. 5a). FACS sorting confirmed the presence of two subpopulations with different CD44 protein levels (CD44^high^ and CD44^low^) in Rh41 cells (Fig. 5b). Being both a receptor for extracellular matrix components and a co-factor for growth factors and cytokines, CD44 is a well-established cancer stem cell marker with great prognostic and therapeutic potentials [31, 32].

We performed three replicates of FACS sorting on Rh41 cells and collected both CD44^high^ and CD44^low^ subpopulations for bulk RNA-seq. Of the 1019 DE genes identified by NBID in the inferred clusters in scRNA-seq, 699 (68.6%) were also detected as DE genes with the same direction in the two subpopulations from the bulk RNA-seq analysis, thus validating CD44 as a cell-surface marker that could be used to separate the two endogenous Rh41 subpopulations. Although MAST identified fewer (448) DE genes, a lower percentage of DE genes (226, 50.4%) were validated in the bulk DE analysis (Additional file 2: Table S13), which demonstrated the superior accuracy and power of NBID in revealing true DE genes. Moreover, among the four established surrogate molecular markers for fusion status in rhabdomyosarcoma samples, namely the upregulation of *TFAP2B*, *MYOG*, and *NOS1*, coupled with the repression of *HMGA2* in fusion positive ARMS [33], both bulk DE analysis of sorted subpopulations and NBID analysis of subpopulations inferred from scRNA-seq revealed repression of *TFAP2B* and *MYOG* as well as upregulation of *HMGA2* in the CD44^high^ subpopulation (Additional file 2: Table S12), suggesting that the CD44^high^ subpopulation represents a less differentiated, stem-like cell subpopulation. The exact mechanism by which the distinct subpopulations develop warrants further investigation.

### Evaluation and control of batch effects

**Fig. 6:**
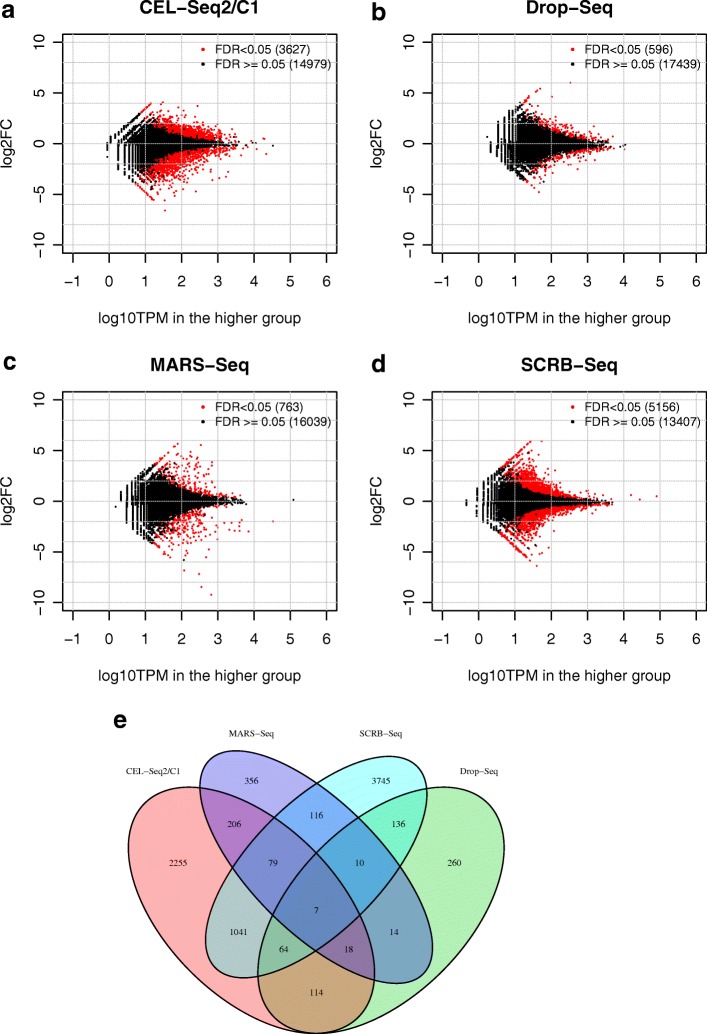
Differential expression analysis of two replicates from Ziegenhain et al. [12]. **a**–**d** The log2 fold change vs the maximal gene log_10_TPM for the two biological replicates. NBID was used for the differential expression analysis of two replicates of each of four UMI-based protocols. The *red dots* indicate genes with FDR < 0.05. **e** *Venn diagram* of DE genes from four UMI-based protocols

Differential gene expression analysis of scRNA-seq data frequently involves data generated in separate batches (e.g. in different lanes or plates in single-cell library construction). This can introduce batch effects (systematic inter-group technical variations that are not relevant to the biological hypothesis being evaluated), which pose a major challenge in high-throughput data analyses [34]. Controlling the batch effects is, therefore, important in order to distinguish true biological differences from technical artifacts [26, 35]. We evaluated batch effects in the two replicates of the four UMI-based protocols used by Ziegenhain et al. [12] (Fig. 6). Although various numbers of DE genes (596–5156, Fig. 6a–d) were detected by these protocols, only seven were common across all protocols (Fig. 6e), consistent with the hypothesis that most apparent DE genes were the result of technical noise. Among the four protocols, CEL-Seq2 and SCRB-Seq had relatively stronger batch effects when compared to DROP-Seq and MARS-Seq; these stronger effects were potentially associated with the higher UMIs captured per cell.

**Fig. 7:**
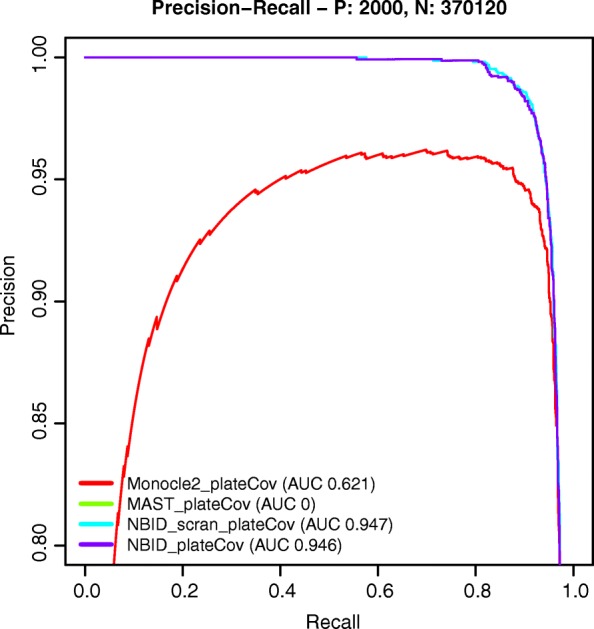
*Precision-recall curves* for selected methods on simulated datasets after adjusting batch variables. P true DE genes, N true non-DE genes

All the evaluated methods except for ROTS allow explicit modeling of technical variations (such as differences in cell-cycle stage and batch effects) as covariates. We evaluated the performance of batch-effect removal by simulating group differences mixed with apparent differences arising from the batch composition, using data generated from CEL-Seq2 (Table 6, Fig. 7) and SCRB-Seq (Additional file 1: Figure S11, Additional file 2: Table S14) by Ziegenhain et al. [12]. Because of the limited sample size in these two datasets, we focused on highly expressed genes (with TPM ≥ 50 in at least one group). Without explicitly modeling the batch effects, all methods showed various levels of FDR inflation. Most of the tested methods (except MAST) reduced the FDR after modeling batch information as a covariate. NBID outperformed Monocle2 and MAST by demonstrating better recovery of true positives with properly controlled FDR.

**Table 6:** FDRs with and without controlling batch covariates

	No filtering				TPM ≥ 50	
Method	FDR	False (n)	DE (n)	FDR	False (n)	DE (n)
Monocle2	**0.279**	37.7	132.4	**0.202**	24.3	118.65
Monocle2_plateCov	**0.100**	10.25	101.3	**0.054**	5.3	96.2
MAST	**0.264**	28.75	74.65	**0.264**	28.75	74.65
MAST_plateCov	**0.268**	28.65	72.85	**0.268**	28.65	72.85
ROTS	**0.258**	36.2	132.1	**0.206**	26.75	122.1
NBID	**0.326**	50.35	146.85	**0.183**	22.9	119.05
NBID_scran	**0.328**	50.45	146.95	**0.200**	25.3	121.45
NBID_plateCov	**0.124**	13.5	108.1	0.048	4.75	99.05
NBID_scran_plateCov	**0.124**	13.45	108	0.048	4.75	98.9

Simulation based on data: CEL-Seq2 (both batch A and batch B) from Ziegenhain et al. [12]. Sample size was 60 (30 cells in each group). In total, Replicate A had 30 cells and Replicate B had 35 cells after QC. Group 1 had 9 cells from Replicate A and 21 cells from Replicate B. Group 2 had 18 cells from Replicate A and 12 cells from Replicate B. Method names with plateCov indicate adjusting the batch covariates. NBID_scran and NBID_scran_plateCov used the size factor computed by scran as the offset instead of the total UMI counts

Bold values indicate FDR > 0.05

Bold and underlined values indicate FDR > 0.1

## Discussion

In the present study, we performed extensive model selection and goodness-of-fit analyses using multiple scRNA-seq datasets and revealed intrinsic distributional differences between the read counts and the UMI counts for the scRNA-seq data. Our analysis suggests that, compared to read counts, UMI counts can be modeled by a simpler distribution. Specifically, the NB model is an adequate model for UMI-count data in the absence of an explicit need to account for dropout events by using zero-inflated models. Data derived from the Smart-Seq protocol in reference [12] deviated slightly from other read-based data, with fewer genes preferring the ZINB model and a lower proportion of NB rejection in the goodness-of-fit test. Although the exact cause of the observation is unknown, our analysis of a different Smart-Seq dataset [23] (Additional file 2: Table S1) resulted in a pattern similar to that seen with other read count-based protocols.

Based on the result of our analysis, we propose a hypothetical model linking the UMI counts and read counts (see Additional file 1: Supplementary methods and Figure S12) that also explains the differences between UMI-count- and read-count-based scatter plots (Fig. 1). The PCR amplification step produces a sharp contrast between the read counts and UMI counts. Whereas the UMI counts follow a Poisson/NB distribution, the read counts—even with a constant multiplication factor (i.e. with no amplification biases)—no longer follow the same Poisson/NB model (see the “Methods” section for more details). The uneven amplification bias (i.e. with transcripts being amplified at different levels) introduces extra deviations from the underneath (simpler) distribution of the UMI counts. Consistent with the hypothesized model, recent studies have shown that inferring approximate transcript counts from the read-count data can significantly improve the analysis efficiency [11].

A few published studies have suggested that NB models are often to be preferred for UMI-based scRNA-seq data [3, 13]. Although we reached the same conclusion, we believe that our design controlled potential technical noises and allowed us to draw a stronger and more valuable conclusion from the extensive evaluation. Grun et al. evaluated the technical noise in the read counts and UMI counts in a relatively small dataset (74 cells) generated by CEL-Seq and concluded that, when compared to the normal and log-normal models, a NB model explained the distribution of more genes [3]. However, the captured UMIs were converted to theoretical transcript-counts (based on the estimated conversion rate) before model fitting. This process could be approximated by the scenario of amplification without biases in our hypothetical model (Additional file 1: Figure S12) and converted transcript counts (in theory) no longer follow a NB model. Consequently, although a NB model explained the distribution of more genes than did the normal and log-normal models, it only accounted for a small fraction of the 11,555 genes analyzed. Recently, Vieth et al. [13] carried out a study that estimated characteristics in 18 UMI-based and 20 read-based scRNA-seq datasets, including those of Ziegenhain et al. [12], that were extensively evaluated in the present study. However, the evaluation of Vieth et al. was based on modeling read counts and UMI counts collected in separate experiments, which inevitably introduced uncontrolled differences between the experiments.

Our design directly compared read counts (before conversion to UMIs) and corresponding UMI counts collected from the same set of cells, enabling us to directly evaluate the effects of PCR amplification in statistical modeling. Moreover, our analysis revealed the necessity of controlling batch differences. Combining the knowledge derived from the extensive modeling with the expected large sample size, we proposed NBID, a novel differential expression analysis algorithm designed for use with UMI-based scRNA-seq data. NBID is based on the negative binomial generalized linear regression (GLM) framework, thus shared similarities with those employed in bulk RNA-seq analysis [36, 37]. The major difference compared to those originally proposed in bulk RNA-seq analysis is that we allow independent group-specific dispersions for each gene based on observations that genes of the same expression level might have different dispersion parameters [3, 7]. Because of the sample size limitation, algorithms proposed in bulk RNA-seq analysis [36, 37] typically pool genes with similar expressions for a robust and smooth estimate of dispersions and assume identical dispersion between groups. NBID exploits the direct benefit of the large sample size in scRNA-seq, which allows group-specific estimates of dispersion for each gene. This difference is analogous to the difference between a t-test assuming equal variance and an unequal variance t-test. Several studies have shown that an unequal variance t-test performs equally well when the underlying group variances are identical but outperforms a t-test assuming equal variance when the group variance are different [38, 39]. Although we focused on comparison to algorithms designed for scRNA-seq in this study, we believe that it will inspire future in-depth evaluations (under various technical scenarios) with additional methods, including those originally proposed for bulk RNA-seq analysis.

Technical variations (e.g. batch effects and variations in the total UMIs captured) are common in scRNA-seq experiments; accounting for these variations is critical to revealing true biological differences in differential expression analysis. As shown in our simulation, many scRNA-seq analysis packages yielded inflated FDRs with technical variations (such as small differences in the total UMIs for the groups), which might result in elevated false positives and/or true positives being masked. In contrast, our analysis indicates that NBID achieved both proper FDR control and better power in revealing real DE genes when compared to previously developed methods for single-cell analysis. Even though only pairwise analyses were considered in the current study, the general form of NBID allows multiple groups to be tested simultaneously, as in the generalized linear model framework.

Differential expression analysis can be used to reveal differences among samples run on separate lanes or plates. However, it should be pointed out that batch effects are expected to overlap with biological differences in this setting. It is better to account for batch effects by proper experimental design, such as by including multiple biological replicates for each group. Another typical application of differential expression analysis is to identify potential biomarkers for inferred cell subpopulations. One potential caveat in this setting is that cells are usually clustered from the same data. Therefore, *p* values or FDR values derived from the differential expression analysis might be overly optimistic. However, the result is still useful for prioritizing potential biomarkers for further validation.

## Conclusions

We have conducted an extensive analysis of multiple scRNA-seq datasets and have concluded that, unlike read counts, UMI counts can be modeled appropriately with the negative binomial model. More complex models, such as zero-inflated negative binomial models, provide no extra gain. Based on the above conclusion, we have proposed a differential expression analysis algorithm that allows independent estimations of dispersion for individual genes within each group. Compared to other recently developed methods, our proposed algorithm achieves proper FDR control and better power for detecting differentially expressed genes in large-scale UMI-count scRNA-seq datasets.

## Methods

### Model selection and testing

We first checked whether the ZINB model was necessary for the UMI counts. This was done by a statistical test comparing the NB and ZINB models for each gene with the null hypothesis that NB fitted the data well. The likelihood ratio statistic was used. We used an FDR level of 0.05 to control the false positives because of the large number of genes tested. For those genes that accepted the NB model, we then checked whether the NB model was necessary by testing the Poisson model versus the NB model, with an FDR level of 0.05. For both NB versus ZINB and Poisson versus NB comparisons, the parameter being tested was on the boundary, and the log likelihood-ratio test statistic follows an equal mixture of 0 mass and a chi-square distribution with 1 degree of freedom under the null hypothesis [40, 41]. The *p* value was calculated based on this mixture distribution, and the FDR was calculated using the Benjamini and Hochberg’s method [42] . To ensure the convergence of fitted NB and ZINB models, we keep only those genes that satisfy L(Poisson) ≤ L(NB) + δ and L(NB) ≤ L(ZINB) + *δ*, where L(M) is the log likelihood of fitted model M, and we set δ to 0.5 to allow some numerical variations in likelihood maximizing. The Poisson model was fitted using the *glm* function in R. Two methods were used to fit a NB model and the one with the higher likelihood was used in the model comparison. The first method was implemented using *glm.nb* in the R package MASS [43]. A grid of initial values 10^[−8, −7, …, 4]^ for *θ* (the reciprocal of the dispersion) was tried, and the largest likelihood was used. The second method was to first fit other parameters related to the mean with an initial dispersion, and then search for the optimal dispersion value to maximize the likelihood given the estimated mean. That method iterated between these two steps until a maximal number of iteration was reached or the change in likelihood was small enough. The ZINB model was fitted using the function *zeroinf* from the R package pscl [44]. To increase the convergence rate, we first fitted a NB model and then used parameters from the NB model as the initial values. For all model comparison, we restricted the comparisons to genes with at least five non-zero cells among all the cells to ensure meaningful expression pattern.

### Goodness-of-fit test

We first down-sampled each cell to the 10% quantile of the total UMI among all the cells so that the gene-count values for each gene would be comparable among cells. The cells corresponding to the lower 10% quantile were not used. The down-sampling was performed by sampling the transcript without replacement, which follows a multivariate hypergeometric distribution. After down-sampling, only genes with a nonzero count in more than five cells were kept. Then the count values were assigned to different intervals (bins). First each unique count value itself forms its own bin and the number of cells falling into each bin was recorded. Staring from the bin of the largest count value, bins with no more than five cells were combined with next bin. The degree of freedom for the Chi-square goodness-of-fit test is *k* − *p* − 1, where *k* is the number of bins and *p* is the number of parameters of the model used. For example, the degree of freedom for the NB model is *k* – 3 and that for the Poisson model is *k* – 2. This procedure filters out genes with expression levels that are too low. For example, genes with count values of only 0 or 1 (two bins) will not be included for testing. However, for these genes, the Poisson or NB model will often result in a very good fit due to the simplicity of the data. In this study, the maximum likelihood estimate of the model parameters were estimated first and then the theoretical counts for individual bins were calculated. We used the R package *fitdistrplus* to plot the empirical pmf/cdf versus the theoretical ones [45].

### Differential expression analysis using the NB model with independent dispersions (NBID)

To simplify the notation, we focus here on one gene. Let us denote the count in cell *i* by *y*_*i*_; then ${y}_i\sim NB\left({n}_i{\mu}_i,{\phi}_{g_i}\right)$, where *n*_*i*_ is the total number of counts for cell *i* and *μ*_*i*_ is the proportion of the gene counts in cell *i*. ${\phi}_{g_i}$ is the dispersion for cell *i* with group label *g*_*i*_, for example, *g*_*i*_ = 0 *or* 1 for two groups. As in generalized linear models, we link the mean proportion to explanatory variables such as group labels; and other potential covariates. Specifically, for two groups, the full model is:

$$ \log \left({n}_i{\mu}_i\right)={\beta}_0+{\beta}_1{g}_i+{\gamma}^T{x}_i, $$

where *β*_0_ is the intercept, *β*_1_ is the group effect size in the log scale, and *γ* is a vector of coefficient for the other covariate vector *x*_*i*_. The likelihood of the observed data under the full model is

$$ L=\prod \limits_{i=1}^mf\left({y}_i|{n}_i{\mu}_i,{\phi}_{g_i}\right), $$

where *m* is the number of cells and $f\left({y}_i|{n}_i{\mu}_i,{\phi}_{g_i}\right)$ is the probability of *y*_*i*_ assuming a NB distribution with mean *n*_*i*_*μ*_*i*_ and dispersion ${\phi}_{g_i}$. Specifically, $f\left(y|\mu, \phi \right)=\frac{\Gamma \left({\phi}^{-1}+y\right)}{y!\Gamma \left({\phi}^{-1}\right)}{\left(\frac{\mu }{\phi^{-1}+\mu}\right)}^y{\left(\frac{\phi^{-1}}{\phi^{-1}+\mu}\right)}^{\phi^{-1}}.$

We note that, here, *n*_*i*_ serves as a normalization factor or size factor, similar to those used in edgeR [36] and DESeq [37]. Alternatively, NBID can accept size factors estimated by other methods, such as scran [25].

We compute the maximum likelihood estimate of the dispersion parameters ${\phi}_{g_i}$ and the coefficients related to the mean by using the R package *nloptr*. To test whether there is a difference between groups, we also fit the null model log(*n*_*i*_*μ*_*i*_) = *β*_0_ + *γ*^*T*^*x* with dispersions estimated from the full model. Finally, a likelihood ratio test is used to compare the reduced model and the full model, which follows a chi-square distribution with one degree of freedom.

### Computing time for NBID in large scale datasets

NBID took 7.5 h in the analysis of naïve T cells versus memory T cell datasets (9850 and 9578 cells, respectively) on an Intel Xeon processor (E5-2670) running Red Hat Enterprise Linux 6 operating system and R 3.3.1.

### Methods evaluated

The methods evaluated and additional details are listed in Additional file 2: Table S4; unless specifically stated otherwise, the default options for each method were used in the evaluation. When there was a need to convert a count value *x* to the log2 scale, log2(*x* + 1) was used for the conversion. The FDR was calculated based on *p* values by Benjamini and Hochberg’s method [42], except for SCDE and ROTS. For SCDE, an adjusted *p* value was used based on the output corrected z-score and assuming a standard normal distribution. For ROTS, we used the FDR output from the package, which was calculated based on the bootstrap resampling.

### Data simulation

To simulate data for differentially expressed analysis, we sampled 1000, 300, or 60 cells from the real UMI-count matrix from memory T cells obtained by GemCode [8], from heterogeneous dendritic cells obtained by MARS-Seq [21], and from whole-intestinal organoids obtained by CEL-Seq [20], respectively. Cells were randomly split into two groups. To create differentially expressed genes, we first ranked genes based on the average count in the second group and chose 50 genes starting with the one having an average UMI count just above *t*. Denoting the fold change by *FC*, we selected another 50 genes starting with the average count just above *FC* × *t*. We then swapped these two sets of genes in their count matrix in the second group. This simulation kept the distribution pattern of the UMI counts unchanged and created differentially expressed genes with certain fold-change levels. In our simulation, we set *FC* and *t* so that the precision-recall curves (power) were in a good range.

To simulate datasets with known batch variables, we sampled different proportions of cells in each replicate to form two groups. Specifically, we sampled nine cells from replicate A and 21 cells from replicate B to form the reference group, 18 cells from replicate A and 22 cells from replicate B to form the other group. We selected DE genes which were not influenced by the apparent difference between replicates. Therefore, when these true DE genes were detected, it was not due to the detection of simulated batch effects. Specifically, we selected DE genes with *p* value > 0.5 from the DE analysis results between the two replicates. We used two platforms in this simulation: CEL-Seq2 and SCRB-Seq, which showed strong batch effects in the DE analysis between two replicates.

### Evaluating FDR and power by using the precision-recall curve

We simulated 100 or 20 replicates for each down-sampling setting. The FDR was calculated for each replicate and then averaged across the replicates to generate the mean FDR. Because a few datasets had running problems with selected competitive methods, replicates on which all methods ran successfully were used in the final analysis. To obtain the power for detecting DE genes, we plotted the precision-recall curve and used the area under the curve (AUC) as the criterion; this was calculated based on all the applicable replicates. Because only the top genes with relatively small estimated FDRs are of interest in a real data analysis, we restricted the comparison to the region where the precision was above 0.8, i.e. the region with FDR ≤ 0.2. This approach was more reasonable than using the full range of the precision-recall curve, even though the result patterns were often similar. This method is also better than using the receiver operating characteristic (ROC) curve as used in some published papers for power comparison because the true negative genes are often the majority; therefore, only the region with very high specificity (so that the FDR can be low) is of interest but the cut-off is not easy to determine with the ROC curve because the specificity is not directly related to the FDR.

#### Rh41 single-cell dataset

The human alveolar rhabdomyosarcoma cell line, Rh41, was cultured in a 5% CO_2_ incubator in a 75-cm^2^ vented flask containing DMEM media supplemented with 10% FBS and 2× glutamine until the cells reached 75% confluence at approximately 3.6×10^6^ cells. The cells were detached from the flask with 7 mL of 1× citrate saline to which 7 mL of DPBS was added followed by centrifugation at 300×G for 7 min. The cells pellet was resuspended in 300 uL of blocking buffer (Rat IgG/PBS) and incubated on ice for 30 min. A total of 50 uL of the cells in blocking buffer were transferred to a separate tube for the isotype control. The cells were washed with 1 mL of staining buffer (5% BSA/PBS) and centrifuged at 300×G for 5 min. The pellet containing approximately 3×10^6^ cells was incubated with Rat IgG2B anti-CD44-Alexa 488 antibody (R&D systems) in staining buffer (15 uL antibody + 135 uL of staining buffer) for 30 min on ice. For the isotype control ~ 600,000 cells were incubated with 5 uL of Rat IgG2B-Alexa488 (R&D systems) + 45 uL of staining buffer for 30 min on ices. After the incubation, both sets of cells were pelleted and washed with 1 mL of staining buffer as described above and resuspended in staining buffer, followed by flow cytometric analysis to identify the fraction of CD44 positive and negative populations.

For the single cell experiment, Rh41 cells were cultured and harvested and washed in DPBS, as described above, and resuspended in PBS/0.2%BSA at a concentration of 1×10^6^ cells/mL. The 10× Genomics single-cell platform performs 3′ gene expression profiling by poly-A selection of mRNA within a single cell, which utilizes a cell barcode and UMIs for each transcript. Single-cell suspensions were loaded onto the Chromium Controller according to their respective cell counts to generate approximately 6000 partitioned single-cell GEMs (Gel Bead-In-EMulsions). The library was prepared using the Chromium Single Cell 3′ v2 Library and Gel Bead Kit (10× Genomics) according to the manufacturers protocol. The cDNA content of each sample after cDNA amplification of 12 cycles was quantified and quality checked by High-Sensitivity DNA chip on a 2100 Bioanalyzer (Agilent Technologies) at a dilution of 1:6. This quantification was used to determine final library amplification cycles in the protocol, which was calculated at 12 cycles. After library quantification and quality check by DNA 1000 chip (Agilent Technologies), samples were diluted to 3.5 nM for loading onto the HiSeq 4000 (Illumina) with a 2 × 75 Paired-end kit using the following read length: 26 bp Read1 (10× cell barcode and UMI), 8 bp i7 Index (sample index), and 98 bp Read2 (insert). An average of 400,000,000 reads per sample was obtained, which translated to roughly 80,000 mean reads per cell, per sample. The Cell Ranger 2.0.1 Single-Cell Software Suite (10× Genomics) was implemented to process the raw sequencing data from the Illumina HiSeq run. This pipeline performed de-multiplexing, alignment (GRCh38/STAR), and barcode processing to generate gene-cell matrices used for downstream analysis.

After matrix generation the ribosomal and mitochondrial related genes were filtered. The subpopulation structure in Rh41 cells was inferred using a novel clustering algorithm developed in house for analyzing large-scale scRNA-seq data (manuscript in preparation). Briefly, it first used singular value decomposition (SVD) to derive latent cellular states from the expression matrix for individual cells. The number of significant cellular states was determined using the Tracy-Widom test on eigenvalues. A modified version of spectral clustering was performed on the significant cellular states of individual cells (cellular states explained by total UMIs were ignored) with a different number of clusters (2–30). The final two-subpopulation structure was determined by the silhouette measure for solutions with different number of clusters.

NBID was used to identify DE genes in the two subpopulations. We further filtered the DE genes by using two thresholds: the average expression level with TPM ≥ 3 in at least one cluster [30], and log2 fold-change ≥ 1.

#### Rh41 bulk RNA-seq dataset

RNA was isolated from the sorted subpopulations using Trizol (Thermo Fisher Scientific) following the manufacture recommendations. RNA libraries were prepared using the Kapa RNA HyperPrep with Riboerase RNA kit (Roche) using the recommended conditions. Briefly, 200 ng of total RNA was used as input for fragmentation, reverse transcription, and second strand synthesis. After clean up, end repair, and A tailing, Nextflex adapters (Bioo Scientific) were ligated to the fragments followed by 12 cycles of PCR amplification on a C1000 (bio-rad). Paired end sequencing was performed (151 bases per read) on a HiSeq 4000 (Illumina). Three replicates were generated. HTSeq [46] was used to produce the count data. edgeR [36] was used for the DE analysis with TMM normalization. Each replicate was coded as a pair of CD44^high^ and CD44^low^ in the analysis.

## Additional files

Additional file 1: This file includes: (1) supplementary methods describing details in single cell quality control and preprocessing, application details of other DE methods, and a statistical model linking UMI and read counts; (2) all supplementary figures. (PDF 2338 kb)

Additional file 2: This file includes all supplementary tables. (XLSX 1530 kb)
