# Supplementary material for: UMI-count modeling and differential expression analysis for single-cell RNA sequencing
Source: Genome Biol. 2018 May 31;19:70. doi: 10.1186/s13059-018-1438-9 (PMC5984373; doi:10.1186/s13059-018-1438-9)
Supplement: Supplementary file 1 — This file includes: (1) supplementary methods describing details in single cell quality control and preprocessing, application details of other DE methods, and a statistical model linking UMI and read counts; (2) all supplementary figures. (PDF 2338 kb) [file 13059_2018_1438_MOESM1_ESM.pdf]

Supplementary methods

Single cell quality control (QC) filtering

We followed the quality control method suggested by the Seurat package. Two plots were generated: one of the number of genes detected against the total UMI count, and one of the proportion of mitochondrial genes against the total UMI count to identify outliers. The thresholds were then decided based on the total UMI count and the proportion of mitochondrial genes' was determined based on a visual inspection of cells. We also did a linear regression between the number of genes detected against the total UMI count both in the log scale, and kept cells within the 95% prediction interval band.

Another QC step used for DE simulation was the removal of few cells with extremely high gene expression for some genes compared to the remaining cells. These outliers might be caused by counting errors or burst of gene expression. First, we standardized the total UMI by converting each UMI count c_{ij} of gene i and cell j to $\frac{c_{ij}}{UMI_j} * median(UMI)$, where UMI_j was the total UMI of cell j and $median(UMI)$ was the median of total UMI across all cells. We then calculated the interquartile IQ of nonzero counts for each gene, and identify the outlier cells with gene expression higher than $q * \max(IQ, 1)$. In all our analysis, we set q to 30. This step usually detected 0 or few cells as outliers.

Data used and preprocessing

Single cell data were downloaded from public resources were summarized in Additional File 2: Table S15. If not specifically stated, we used the downloaded gene-cell count matrices without further filtering or normalization.

Single cell data from Ziegenhain et al. 2017 [1] using 6 protocols

For all analysis, we used the data described in Ziegenhain et al. [1]. The final count matrices were used. We removed ERCC RNAs counts from the count matrix therefore focusing on endogenous mRNA counts. In DE analysis in two replicates and DE simulations with batch effects, outlier detection was applied to remove several cells with extremely high gene expressions for some genes.

Naïve T cell, memory T cell From Zheng et al. 2017 [2]

We first applied QC as described above. For simulations, outlier detection were applied, no cells were removed.

Dataset from Grun et al. 2015 [3]

The YFPpos and Whole_Organoid_Replicate_2 data sets were used in our analysis. We removed ERCC RNA counts first and then did QC described above. For simulations based on the plate I5d, few outlier cells with very high gene expressions for some genes were removed.

Dataset from Jatin et al. 2014 [4]

We first removed ERCC genes and 3 genes with different number of cells. Then we extract the CD11c+ and CD11c+(2hr_LPS) cells. For each data set, we applied QC described above. For simulations based on CD11c+ cells, we applied outlier detection, no cells were removed.

Dataset from Klein et al. 2015 [5]

The downloaded count matrix was used directly.

Dataset from Islam et al. 2011 [6]

We first removed spike in RNAs and use the 48 ESC cells for our analysis.

Dataset from Scialdone et al. 2015 [7]

We kept single cells with quality OK and removed ERCC genes.

Application of other DE Methods

Monocle2: We followed the analysis code in the supplementary file of the Monocle2 paper https://www.nature.com/articles/nmeth.4150#s4. For the UMI count, it requires the full count matrix. It has its own function to estimate the size factors for normalization.

SCDE: We followed the link http://hms-dbmi.github.io/scde/diffexp.html. SCDE requires the count matrix as the input.

MAST: We followed the link https://www.bioconductor.org/help/course-materials/2016/BioC2016/ConcurrentWorkshops2/McDavid/MAITAnalysis.html. Specifically we used log2 scale TPM as the input and used adaptive thresholding used in the tutorial.

ROTS: We followed the link: https://bioconductor.org/packages/release/bioc/vignettes/ROTS/inst/doc/ROTS.pdf. Specifically, we used TMM normalization as recommended by the package.

Seurat: We follow the link http://satijalab.org/seurat/pbmc3k_tutorial_1_4.html. The total UMI is regressed out except for the Poisson or negative binomial model. For Poisson or negative binomial model, the total UMI is incorporated into the DE model.

A statistical model linking the UMI count and read count of single cells

Our proposed model illustrates the relation and difference between the UMI count and read count (Additional file 1: Fig. S12), being motivated by the work of Qiu et al. [8]. Because the efficiency of the reverse transcription from the original mRNA to cDNA in scRNA-seq is low,

e.g., less than ~10% [9, 10], the cDNA count or UMI count for each cell can be modelled as a sampling process from the pool of mRNA. When the mRNA proportions are the same among cells (i.e., there is no biological variation) and the only technique variation is in the sampling depth, e.g., the total UMI per cell, the gene count follows a multinomial distribution or approximately Poisson distribution given the small gene proportions. This is the simplest distribution with which to model the UMI count when considering different sampling depths. When there are some moderate heterogeneity, i.e., when the mRNA proportions vary across cells, the negative binomial (NB) model is often used. This is the case if we assume that the mRNA proportion of a given gene in each cell follows a gamma distribution. For the read count, we need to consider the additional amplification process, which is a multiplication of the captured cDNA. In the ideal case, the multiplication factor is a constant across all cells and genes. In practice, however, this multiplication factor is likely to differ among cells and genes, thereby introducing more complexity. The final sequencing read count can be modeled as a multinomial sampling process depending on the final total sequence reads and the proportion of the amplified cDNA.

We can formalize the above model into statistical formulae. We do not consider spike-in RNAs here. Let R_{ij} be the mRNA count, with the subscripts indicating cell i and gene j, then the total RNA count for the cell i is $N_i = \sum_j R_{ij}$. Some mRNAs may be degraded or lost during cell separation or lysis. The available mRNA before reverse transcription can be modeled as follows:

$$X_{ij} = \alpha_i R_{ij},$$

where α_i is cell specific and unknown. Because reverse transcription from mRNA to cDNA is a low-efficiency process in single cells, it can be modeled as a sampling process using a multinomial distribution:

$$Y_{ij} = multinomial\left(\frac{X_{ij}}{\sum_j X_{ij}}, n_i\right) = multinomial\left(\frac{R_{ij}}{\sum_j R_{ij}}, n_i\right),$$

where n_i is the cDNA count, and $n_i = \theta_i \sum_j X_{ij} = \alpha_i \theta_i N_i$, θ_i represents the reverse transcription efficiency for cell i. Here, we assume that the efficiency is only cell specific but is the same for all genes. Because the cDNA count per gene is usually small and the total cDNA count in a cell is large, Y_{ij} can be approximated using a Poisson distribution with mean $n_i p_{ij}$, where $p_{ij} = \frac{R_{ij}}{\sum_j R_{ij}}$, the proportion of each gene transcript. Different cells will exhibit some degree of variation in gene proportions even for homogeneous cell populations. If we assume that p_{ij} follows a gamma distribution parameterized as $gamma(scale = \frac{1}{\phi_j}, rate = \frac{1}{\phi_j p_j})$, where p_j and ϕ_j are the mean proportion and dispersion of gene j, respectively, then the cDNA count Y_{ij} follows a negative binomial distribution $NB(n_i p_j, \phi_j)$. Here, we assume that the UMI count catches all available cDNA with sufficient sequencing depth.

For the amplification process, because it is likely to be specific for both cells and genes, we use a general model for the amplified cDNA count as follows:

$$Z_{ij} = \gamma_{ij} Y_{ij},$$

where γ_{ij} is the amplification parameter for cell i and gene j. This could be simplified to a constant or could be only cell or gene specific with more knowledge of the amplification process.

The last step is sequencing. The read count C_{ij} can be modeled as a multinomial sampling process given the total reads:

$$C_{ij} = multinomial(\frac{Z_{ij}}{\sum_j Z_{ij}}, S_i),$$

where S_i is the total reads for cell i. Because the reverse-transcribed cDNA Y_{ij} has many 0s, resulting in many 0s in Z_{ij} even after amplification and many zero reads in C_{ij}, modeling C_{ij} will be more complex than modeling Y_{ij} directly. For example, when Y_{ij} has three distinct values 0, 1, or 2, for a pool of cells, this is likely to result in three clusters for the read counts, corresponding to Y_{ij} being 0, 1, or 2, respectively. In this case, even a ZINB model might not model the read count well.

Our model differs from that of Qiu [8] in the following respects. The cDNA count before amplification is modeled as a sampling process, and we explicitly separate the amplification process and the sequencing process. The first difference explains the massive 0 counts in the scRNA-seq data matrix. The second illustrates the complication involved in the amplification process, as well as the advantage of using UMI to avoid this complication.

In the above model we assume that the UMI count captures all the cDNAs. This is probably the case when sufficient sequencing depth is used. Otherwise the UMI count is again a sampling of the cDNA count, which can be combined with the sampling process of reverse transcription with decreased capture efficiency and the subsequent modeling will be the same.

References

1. Ziegenhain C, Vieth B, Parekh S, Reinius B, Guillaumet-Adkins A, Smets M, Leonhardt H, Heyn H, Hellmann I, Enard W: **Comparative Analysis of Single-Cell RNA Sequencing Methods.** *Mol Cell* 2017, **65:**631-643 e634.

2. Zheng GX, Terry JM, Belgrader P, Ryvkin P, Bent ZW, Wilson R, Ziraldo SB, Wheeler TD, McDermott GP, Zhu J, et al: **Massively parallel digital transcriptional profiling of single cells.** *Nat Commun* 2017, **8:**14049.
3. Grun D, Lyubimova A, Kester L, Wiebrands K, Basak O, Sasaki N, Clevers H, van Oudenaarden A: **Single-cell messenger RNA sequencing reveals rare intestinal cell types.** *Nature* 2015, **525:**251-255.
4. Jaitin DA, Kenigsberg E, Keren-Shaul H, Elefant N, Paul F, Zaretsky I, Mildner A, Cohen N, Jung S, Tanay A, Amit I: **Massively parallel single-cell RNA-seq for marker-free decomposition of tissues into cell types.** *Science* 2014, **343:**776-779.
5. Klein AM, Mazutis L, Akartuna I, Tallapragada N, Veres A, Li V, Peshkin L, Weitz DA, Kirschner MW: **Droplet barcoding for single-cell transcriptomics applied to embryonic stem cells.** *Cell* 2015, **161:**1187-1201.
6. Islam S, Kjallquist U, Moliner A, Zajac P, Fan JB, Lonnerberg P, Linnarsson S: **Characterization of the single-cell transcriptional landscape by highly multiplex RNA-seq.** *Genome Res* 2011, **21:**1160-1167.
7. Scialdone A, Natarajan KN, Saraiva LR, Proserpio V, Teichmann SA, Stegle O, Marioni JC, Buettner F: **Computational assignment of cell-cycle stage from single-cell transcriptome data.** *Methods* 2015, **85:**54-61.
8. Qiu X, Hill A, Packer J, Lin D, Ma YA, Trapnell C: **Single-cell mRNA quantification and differential analysis with Census.** *Nat Methods* 2017, **14:**309-315.
9. Marinov GK, Williams BA, McCue K, Schroth GP, Gertz J, Myers RM, Wold BJ: **From single-cell to cell-pool transcriptomes: stochasticity in gene expression and RNA splicing.** *Genome Res* 2014, **24:**496-510.
10. Grun D, Kester L, van Oudenaarden A: **Validation of noise models for single-cell transcriptomics.** *Nat Methods* 2014, **11:**637-640.

Supplementary figure legends

Figure S1. Results of model comparison using both UMI and read counts. The circle graphs show the proportion of genes with the selected models for six different protocols using hypothesis testing. A significant portion of genes favored the simple Poisson model when UMI counts were used **(a)**, but the portion favoring the Poisson model sharply dropped when the read counts were used **(b)**. Also the zero-inflated negative binomial (ZINB) model was non-negligible for six of the twelve read count-based datasets.

Figure S2. Two clusters in Rh41 cells. The bar at the top indicates the cluster membership. The lower panel shows the color coded dissimilarity matrix among cells within and between clusters.

Figure S3. Goodness of fit using the negative binomial distribution for memory T cells (Tm). **a**, **c**, and **e** are the empirical and theoretical probability mass functions (pmfs). **b**, **c**, and **f** are the empirical and theoretical cumulative distribution functions (cdfs). Three genes with different significance levels are shown.

Figure S4. Goodness of fit using the negative binomial distribution for mouse Rh41 combined cells. **a**, **c**, and **e** are the empirical and theoretical probability mass functions (pmfs). **b**, **c**, and **f** are the empirical and theoretical cumulative distribution functions (cdfs). Three genes with different significance levels are shown.

Figure S5. Precision recall curves for selected methods on simulated data set based on memory T cells from Zheng et al. with fold change 1.7. The starting average count of DE genes from the reference group was 0.2. Sample size was 1000 (500 cells per group). P: true DE genes, N: true non-DE genes.

Figure S6. Precision recall curves for selected methods on simulated data set based on memory T cells from Zheng et al. with fold change 2. The starting average count of DE genes from the reference group was 0.1. Sample size was 1000 (500 cells per group). P: true DE genes, N: true non-DE genes.

Figure S7. Precision recall curves for selected methods on simulated data set based on memory T cells from Zheng et al. with fold change 3. The starting average count of DE genes from the reference group was 0.05. Sample size was 1000 (500 cells per group). P: true DE genes, N: true non-DE genes.

Figure S8. Precision recall curves for selected methods on simulated data set based on dendritic (CD11c+) cells from mouse spleen from Jaitin et al. with fold change 1.8. The starting average count of DE genes from the reference group was 0.4. Sample size was 300 (150 cells per group). P: true DE genes, N: true non-DE genes.

Figure S9. Precision recall curves for selected methods on simulated data set based on YFP positive cells from plate I5d from Grun et al. with fold change 4. The starting average count of DE genes from the reference group was 0.8. Sample size was 60 (30 cells per group). P: true DE genes, N: true non-DE genes.

Figure S10. Density plots of the expression patterns of selected DE genes in memory and naïve T cells. Two density plots are shown for each gene, with the left one showing the global density plot and the right one showing the zoomed-in density in the nonzero TPM regions. The 1st column shows known DE genes between the two cell types. *LGALS1* was detected as a DE gene by NBID, MAST and ROTS. *IFNG* is detected as a DE gene by NBID and MAST. *CXCR5* is detected as a DE gene by NBID only, *TOX* is not detected as a DE gene by any of the above

three methods. The y-axis is the TPM / 100 plus 1 and then converted to log10 scale. The 2nd and 3rd column show DE genes detected only by NBID in 8 different FDR percentile ranges and TPM > 50 in at least one population.

Figure S11. Precision recall curve for selected methods after adjusting batch variables on simulated data sets based on the SCRB-Seq replicates. P: true DE genes, N: true non-DE genes.

Figure S12. A hypothetical model illustrating the generation of UMI counts and read counts. The counts in the figure are hypothetical and illustrate the effects of each processing step.

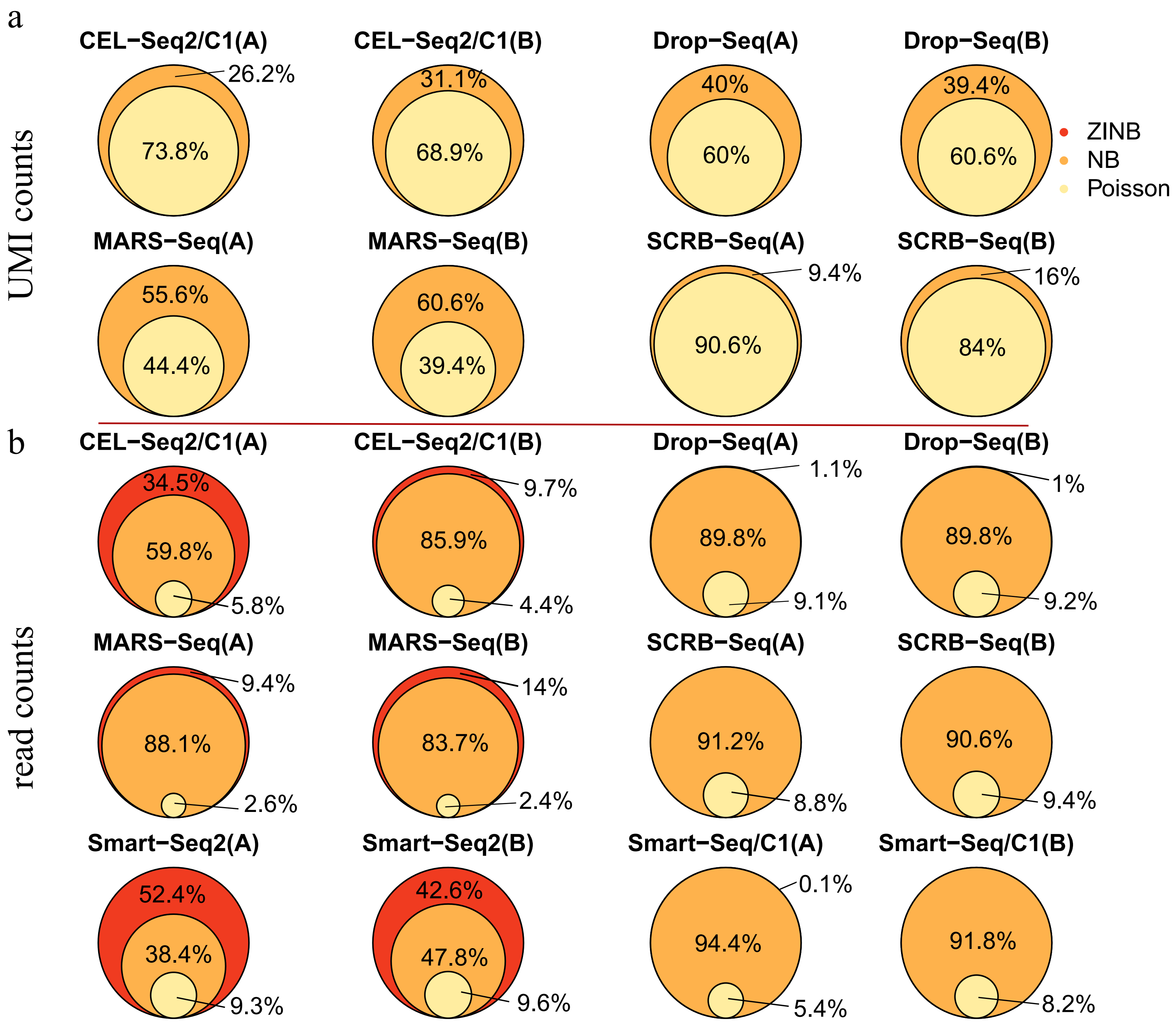

Figure S1. Results of model comparison using both UMI and read counts. The circle graphs show the proportion of genes with the selected models for six different protocols using hypothesis testing. A significant portion of genes favored the simple Poisson model when UMI counts were used (a), but the portion favoring the Poisson model sharply dropped when the read counts were used (b). Also the zero-inflated negative binomial (ZINB) model was non-negligible for six of the twelve read count-based datasets.

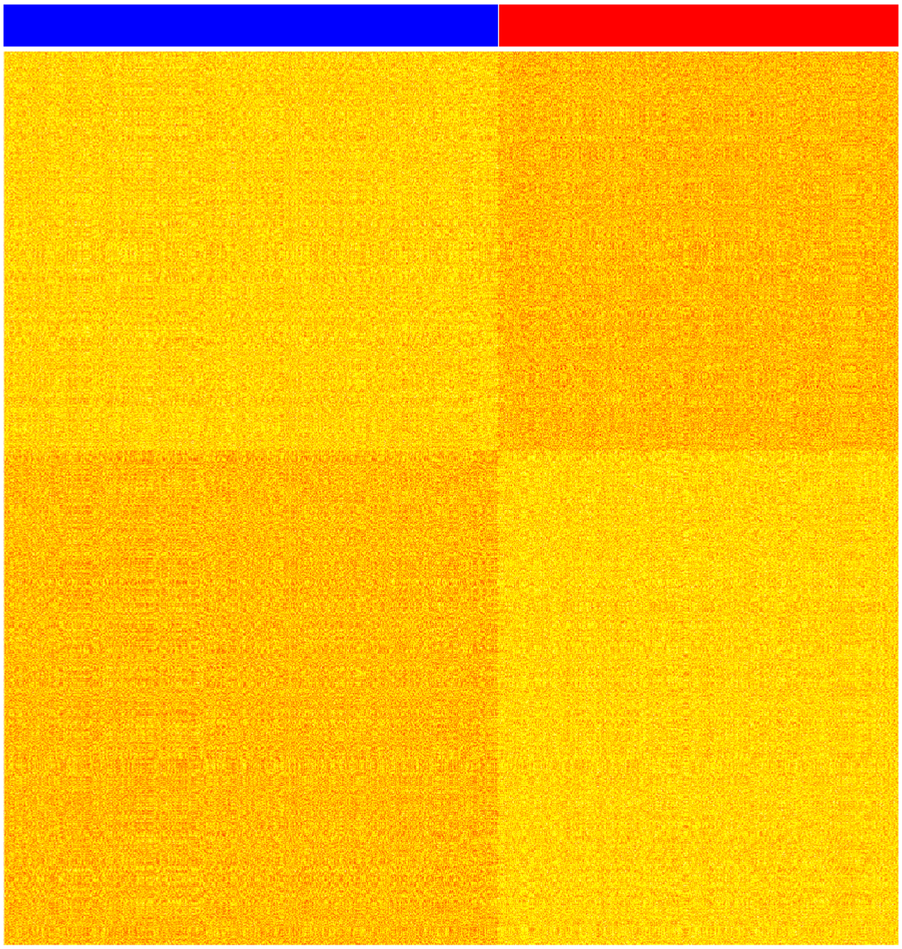

Figure S2. Two clusters in Rh41 cells. The top bar indicates the cluster membership. The lower panel shows the color coded dissimilarity matrix among cells within and between clusters.

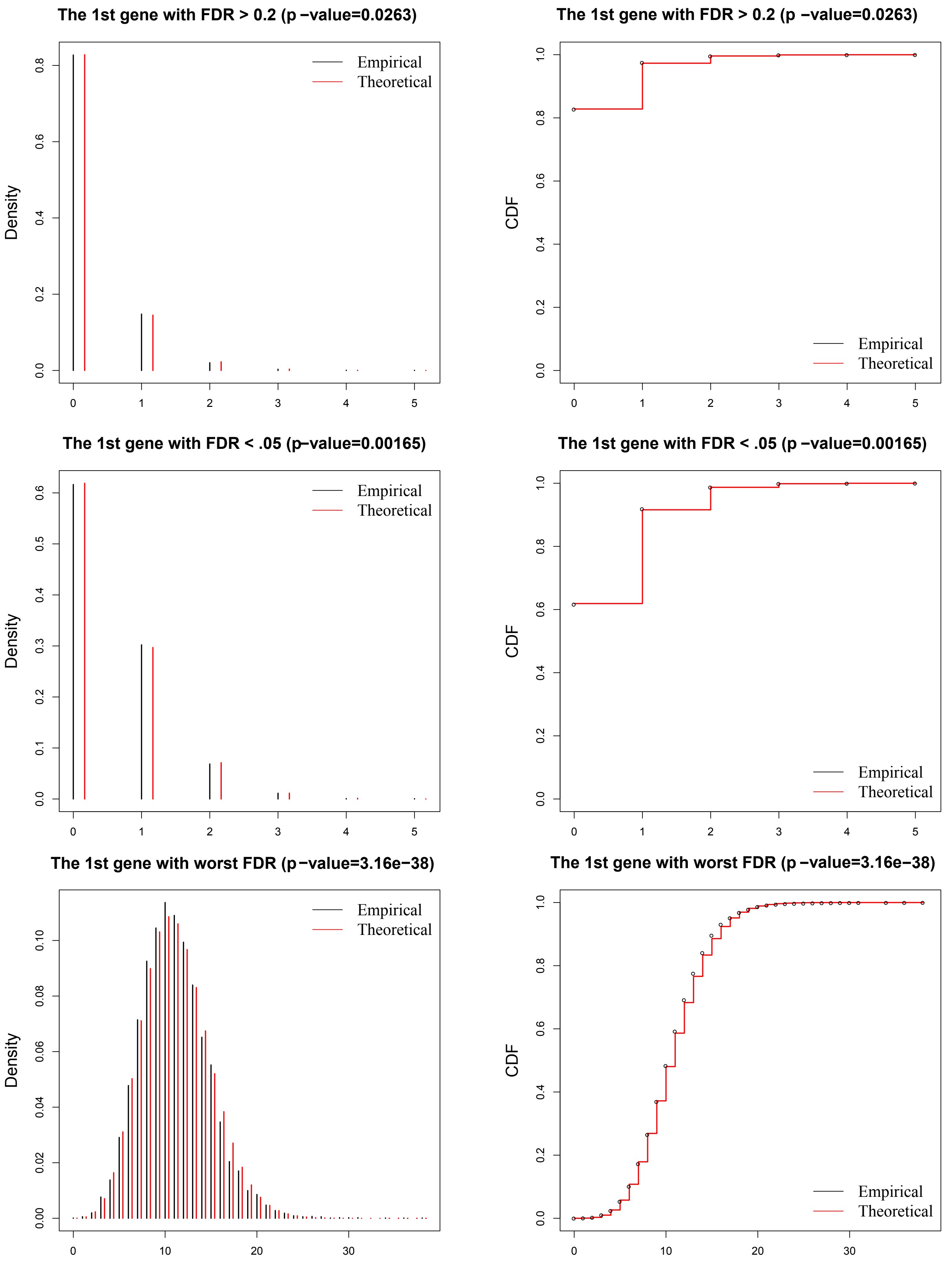

Figure S3. Goodness of fit using the negative binomial distribution for memory T cells (Tm). a, c, e are the empirical and theoretical probability mass functions (pmf). b, c, f are the empirical and theoretical cumulative distribution functions (cdf). Three genes of different significance levels are shown in each row.

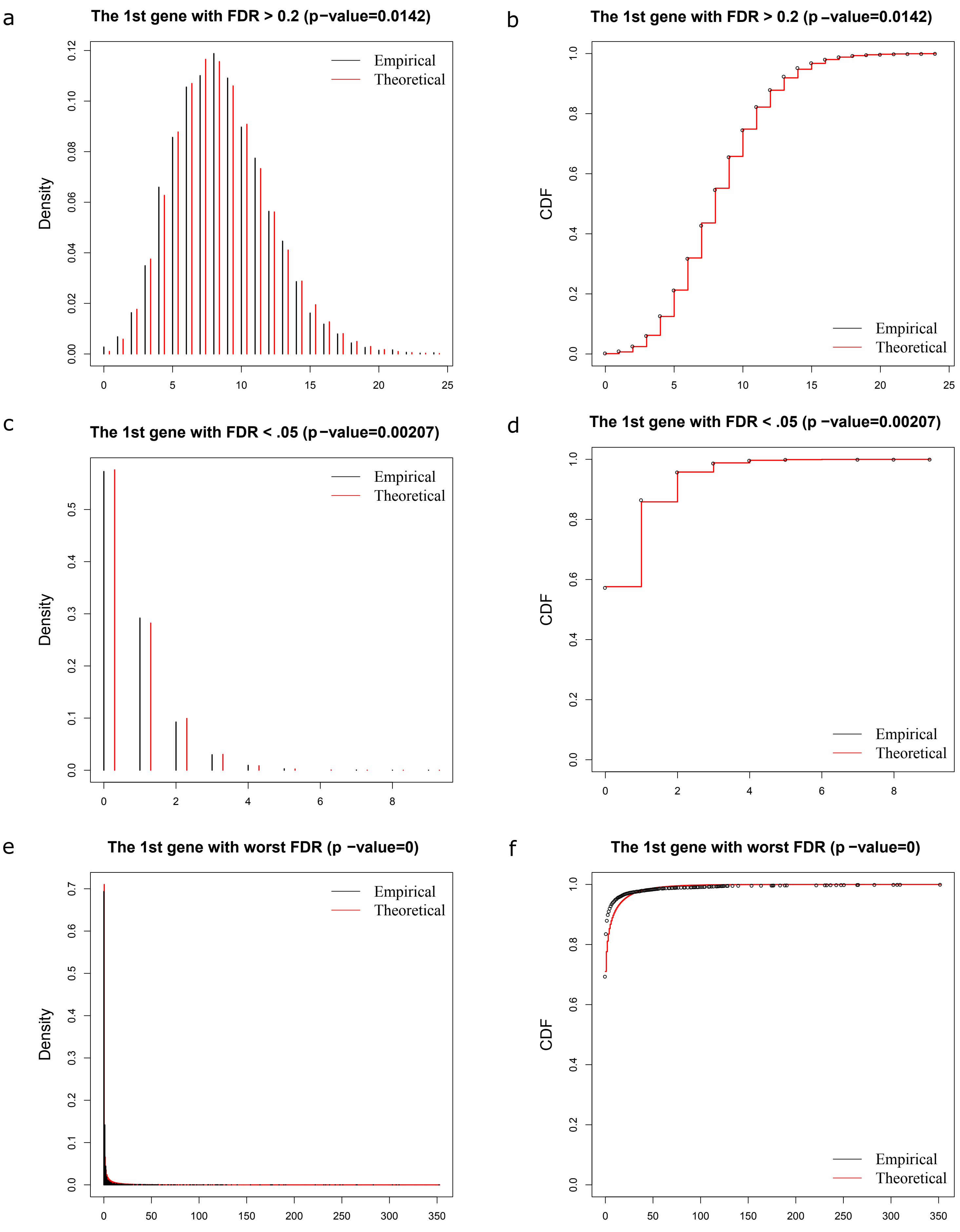

Figure S4. Goodness of fit using the negative binomial distribution for mouse Rh41 combined cells. a, c, e are the empirical and theoretical probability mass functions (pmf). b, c, f are the empirical and theoretical cumulative distribution functions (cdf). Three genes of different significance levels are shown in each row.

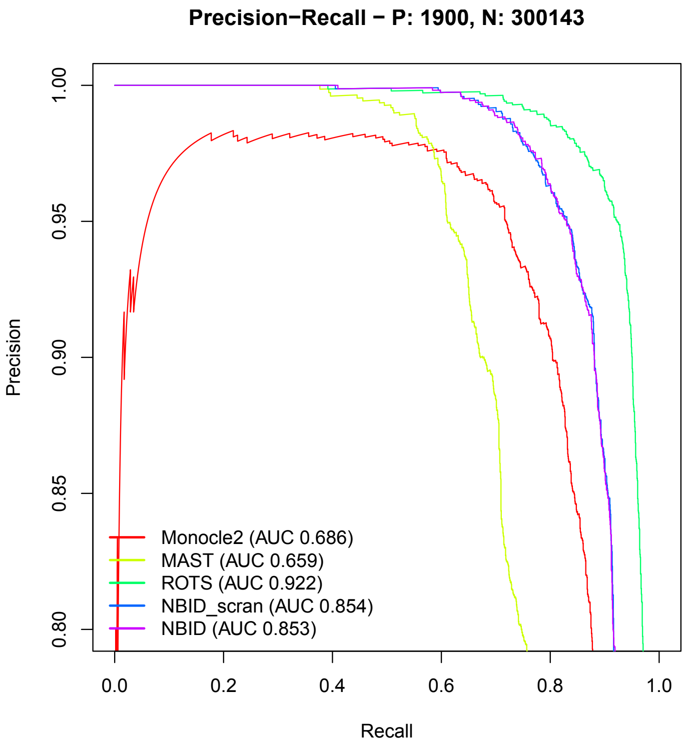

(a) no UMI differences

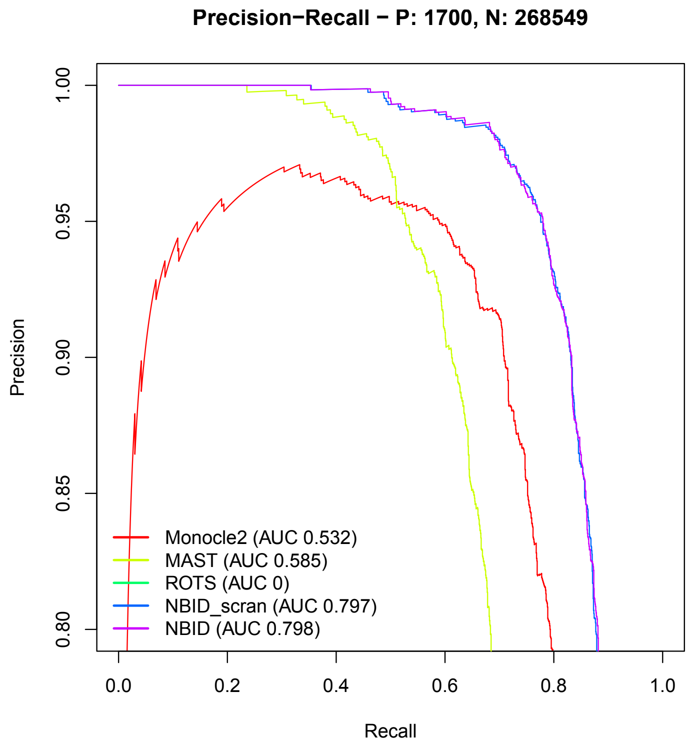

(b) mild UMI differences

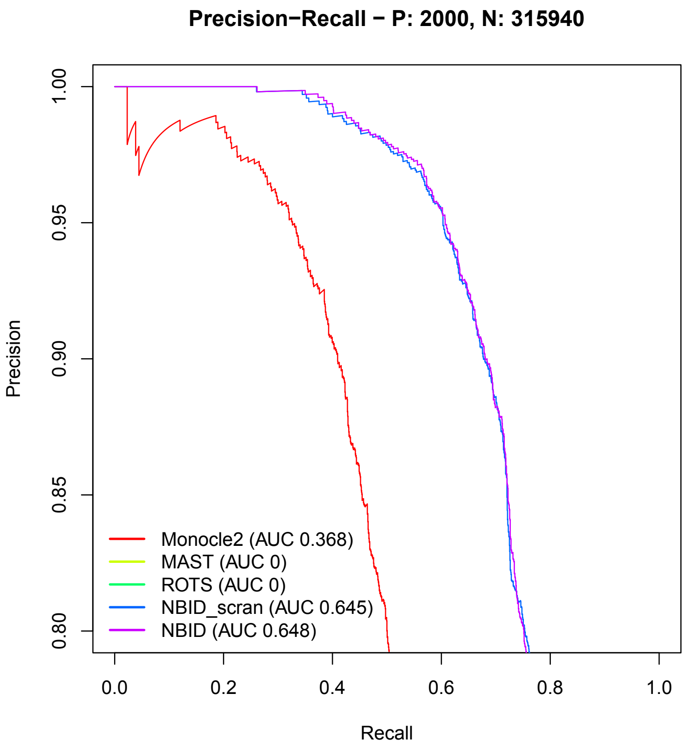

(c) intermediate UMI differences

Figure S5. Precision recall curves for selected methods on simulated data set based on memory T cells from Zheng et al. [8] with fold change 1.7. The starting average count of DE genes from the reference group was 0.2. Sample size was 1000 (500 cells per group). P: true DE genes, N: true non-DE genes.

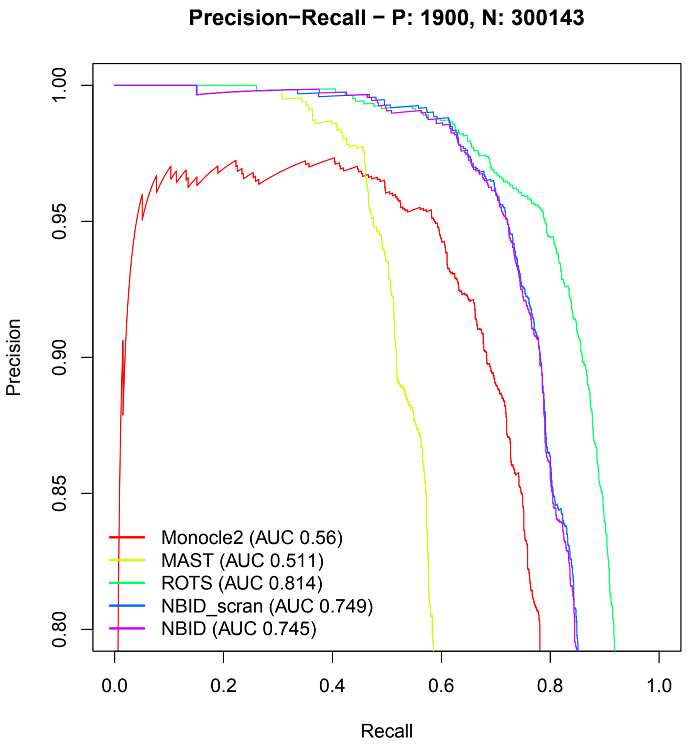

(a) no UMI dif erences

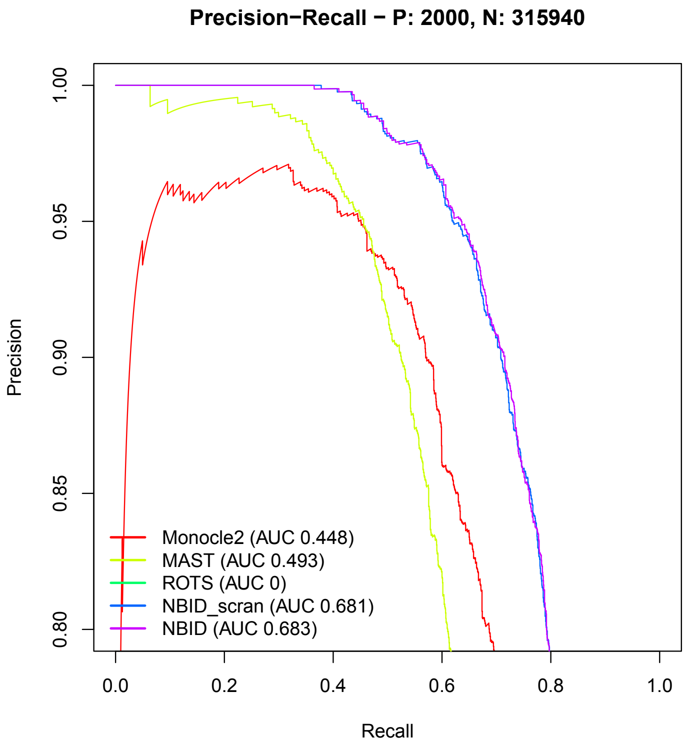

(b) mild UMI dif erences

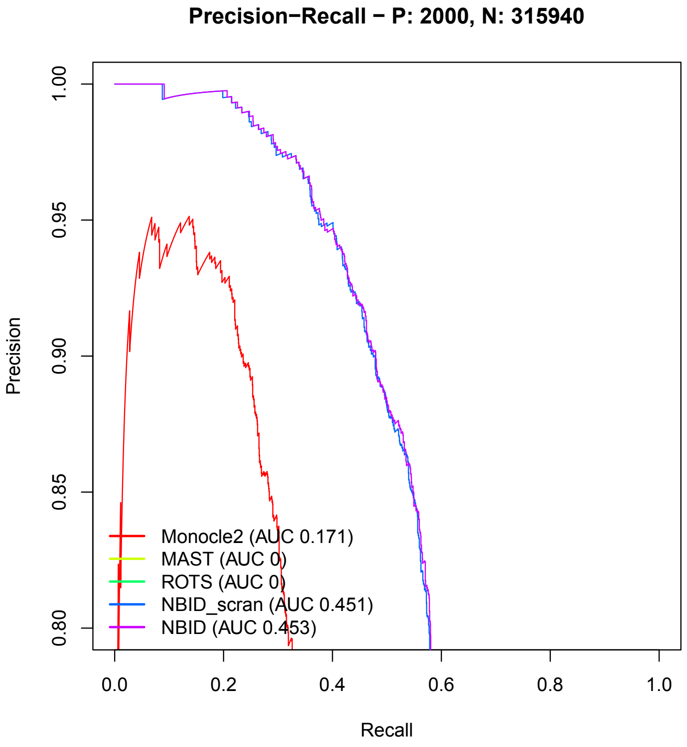

(c) intermediate UMI dif erences

Figure S6. Precision recall curves for selected methods on simulated data set based on memory T cells from Zheng et al. [8] with fold change 2. The starting average count of DE genes from the reference group was 0.1. Sample size was 1000 (500 cells per group). P: true DE genes, N: true non-DE genes.

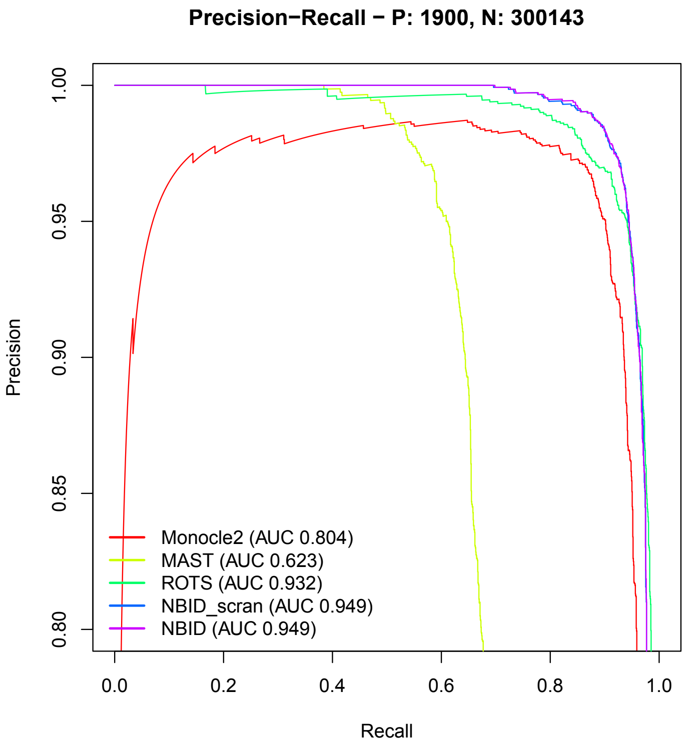

(a) no UMI dif erences

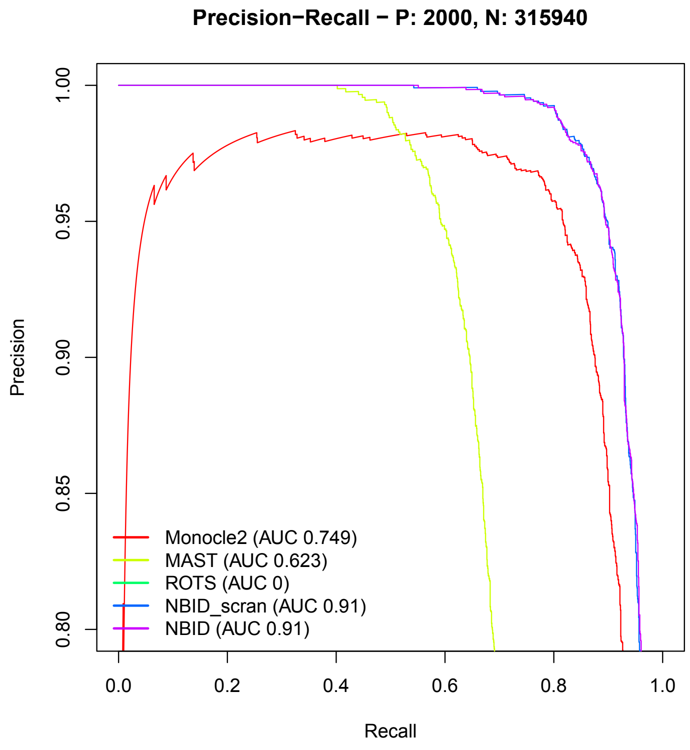

(b) mild UMI dif erences

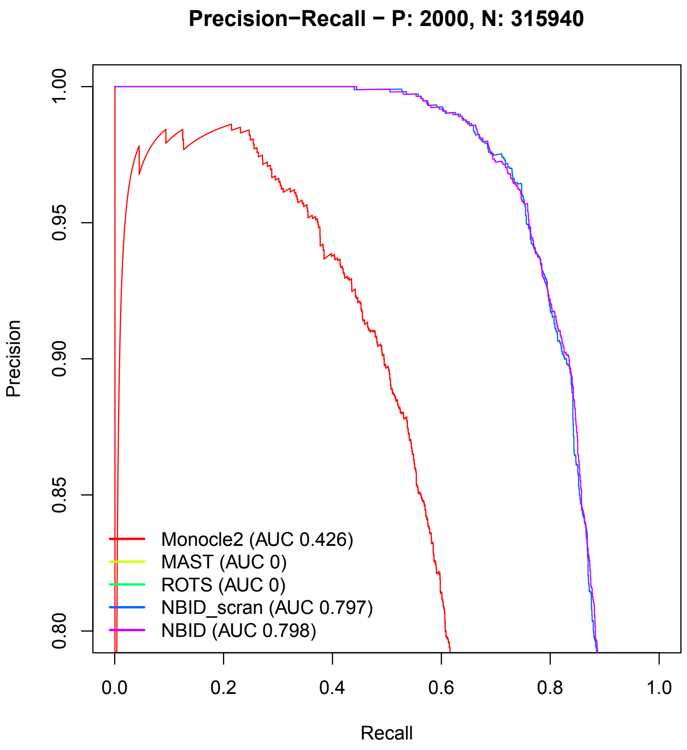

(c) intermediate UMI dif erences

Figure S7. Precision recall curves for selected methods on simulated data set based on memory T cells from Zheng et al. [8] with fold change 3. The starting average count of DE genes from the reference group was 0.05. Sample size was 1000 (500 cells per group). P: true DE genes, N: true non-DE genes.

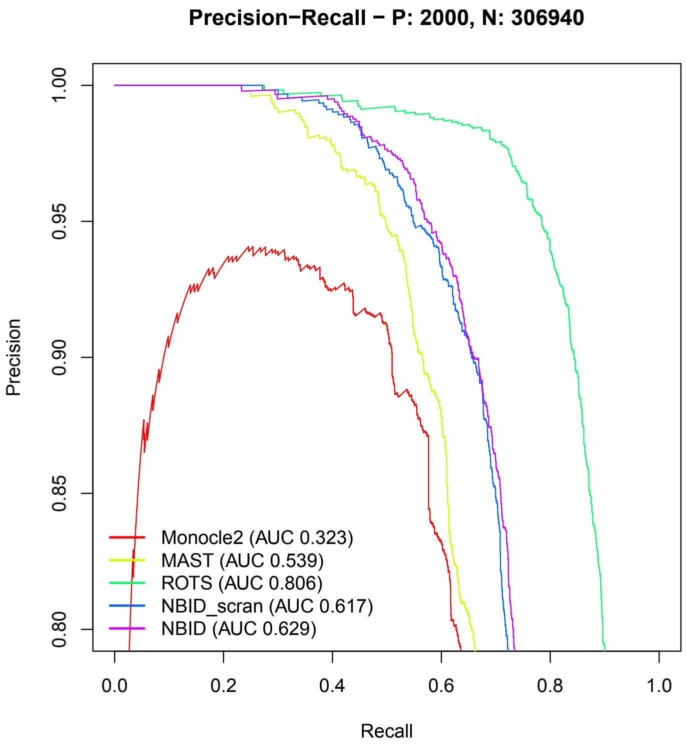

(a) no UMI differences

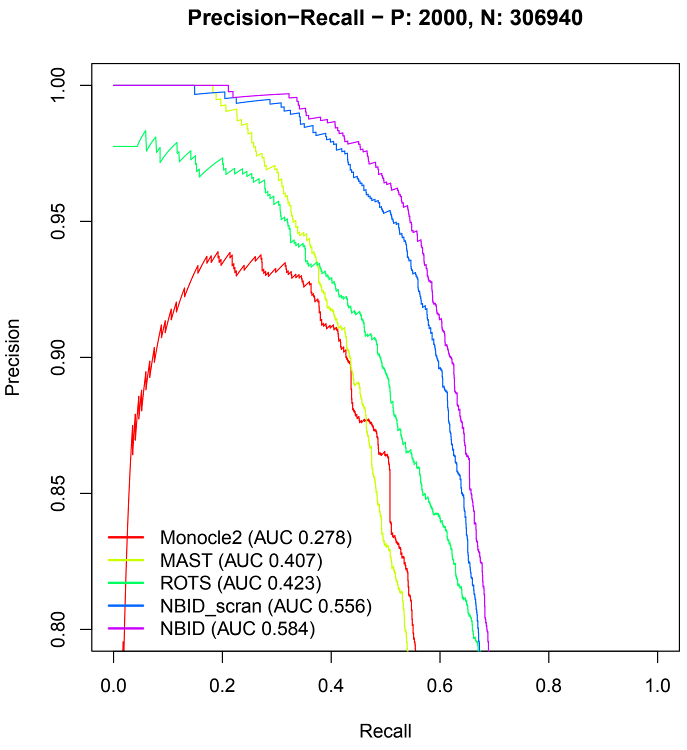

(b) mild UMI differences

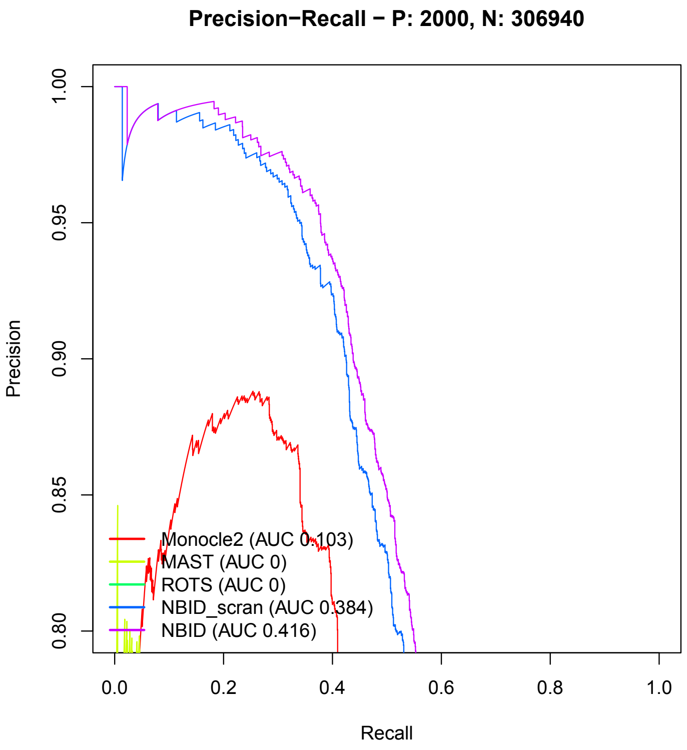

(c) intermediate UMI differences

Figure S8. Precision recall curves for selected methods on simulated data set based on dendritic (CD11c+) cells from mouse spleen from Jaitin et al. [21] with fold change 1.8. The starting average count of DE genes from the reference group was 0.4. Sample size was 300 (150 cells per group). P: true DE genes, N: true non-DE genes.

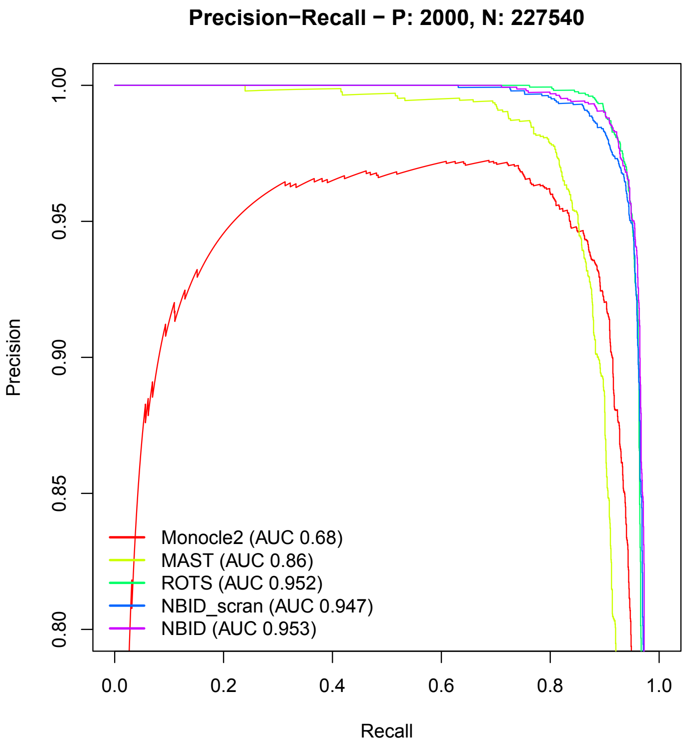

(a) no UMI dif erences

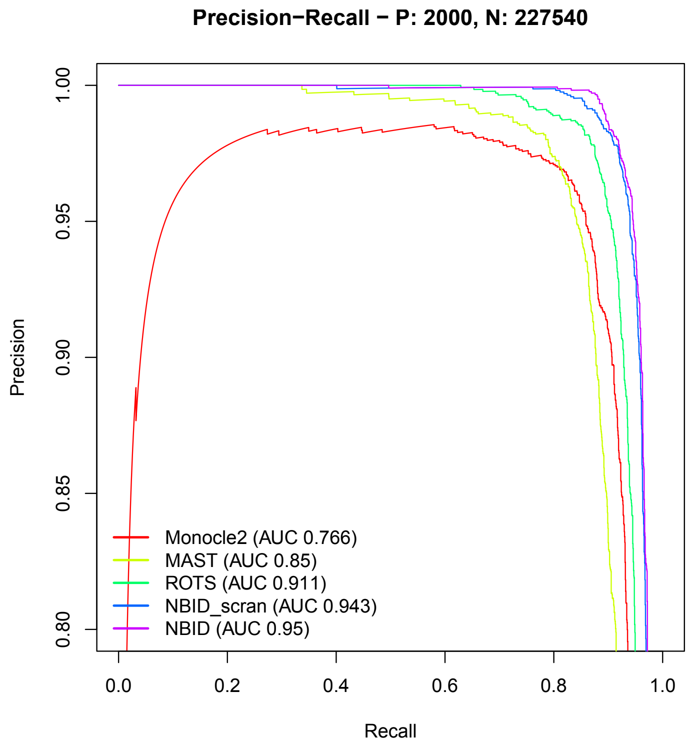

(b) mild UMI dif erences

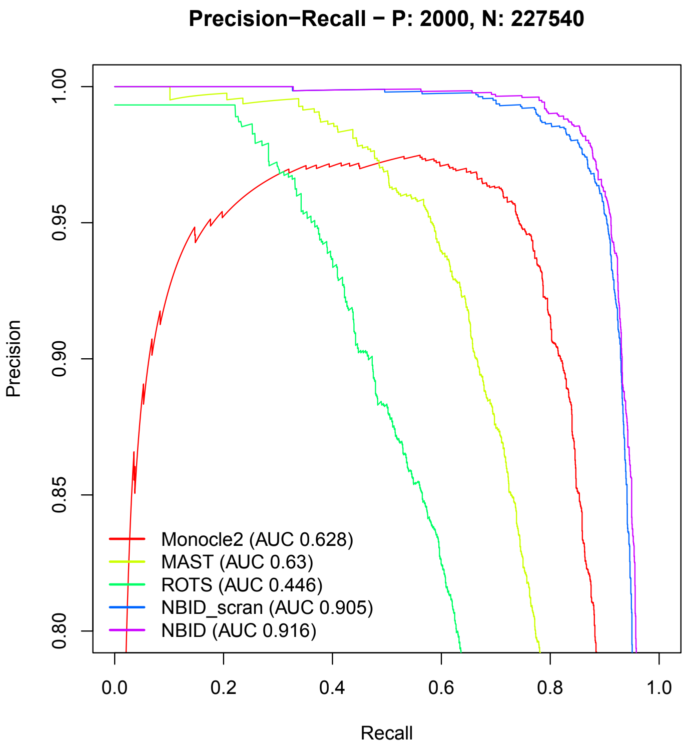

(c) intermediate UMI dif erences

Figure S9. Precision recall curves for selected methods on simulated data set based on YFP positive cells from plate I5d from Grun et al. [20] with fold change 4. The starting average count of DE genes from the reference group was 0.8. Sample size was 60 (30 cells per group). P: true DE genes, N: true non-DE genes.

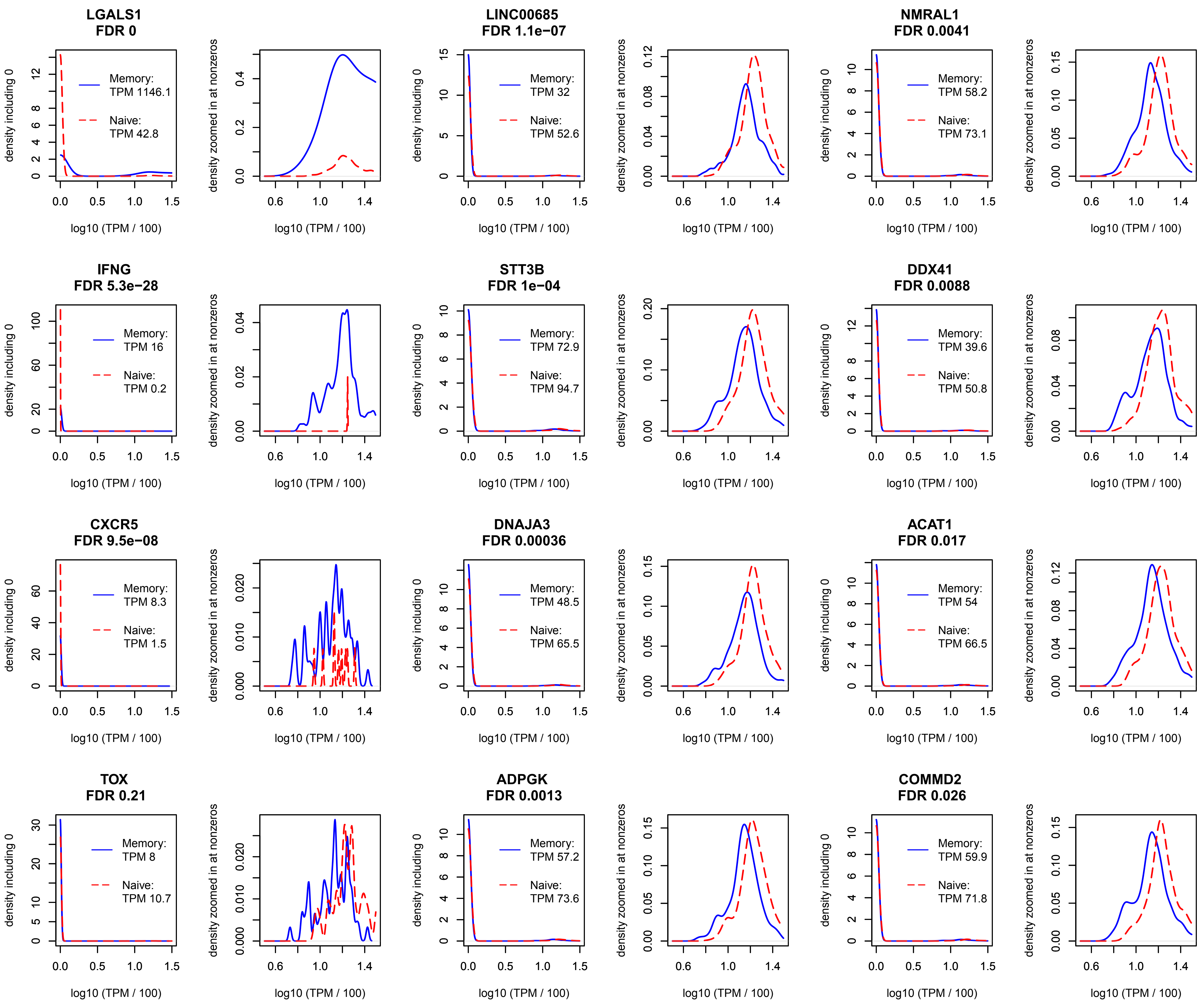

Figure S10. Density plots of the expression patterns of selected DE genes in memory and naïve T cells. Two density plots are shown for each gene, with the left one showing the global density plot and the right one showing the zoomed-in density in the nonzero TPM regions. The 1st column shows known DE genes between the two cell types. LGALS1 was detected as a DE gene by NBID, MAST and ROTS. IFNG is detected as a DE gene by NBID and MAST. CXCR5 is detected as a DE gene by NBID only, TOX is not detected as a DE gene by any of the above three methods. The y-axis is the TPM / 100 plus 1 and then converted to log10 scale. The 2nd and 3rd column show DE genes detected only by NBID in 8 different FDR percentile ranges and TPM > 50 in at least one population.

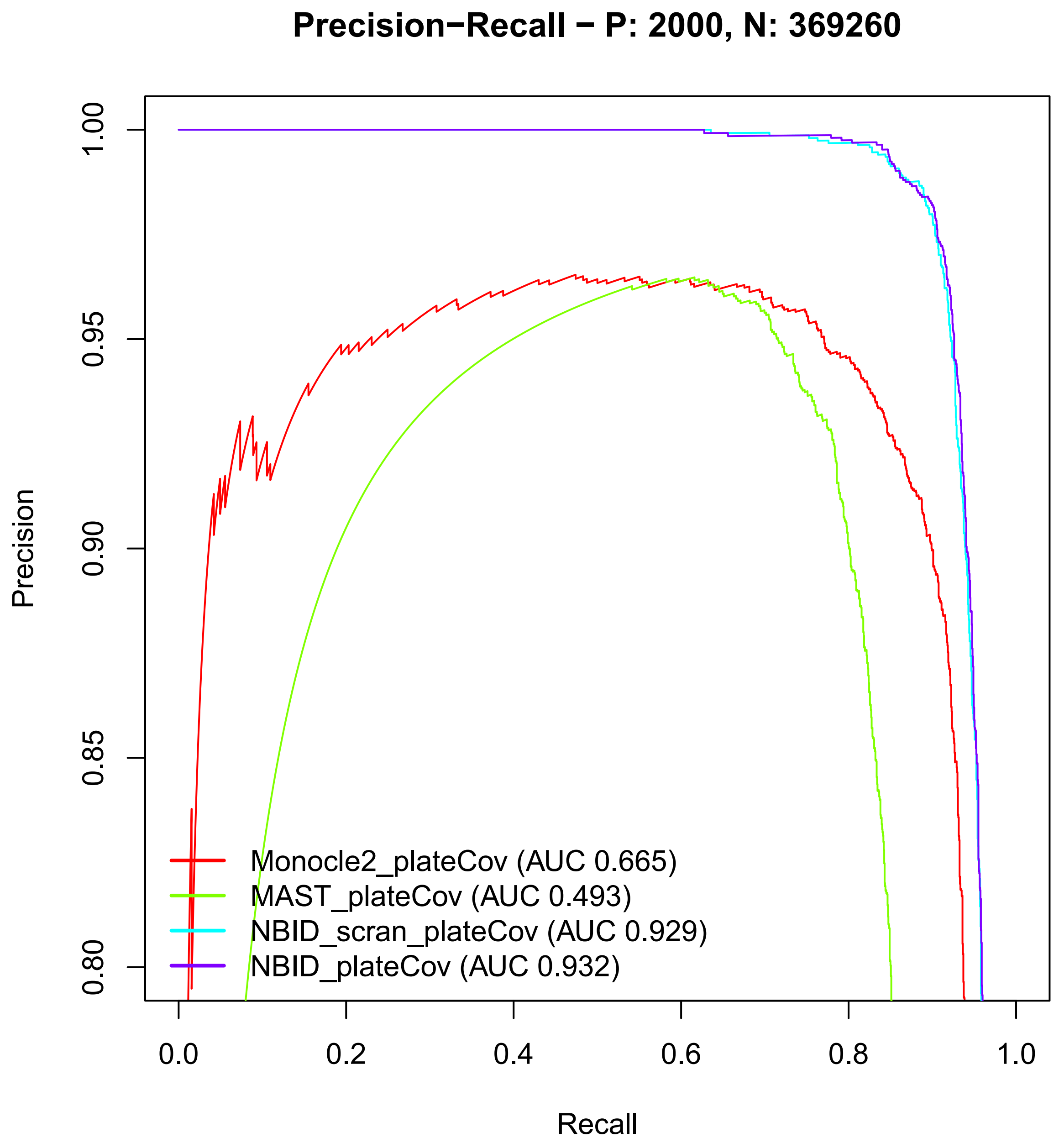

Figure S11. Precision recall curve for selected methods after adjusting batch variables on simulated data sets based on the SCRB-Seq replicates. P: true DE genes, N: true non-DE genes.

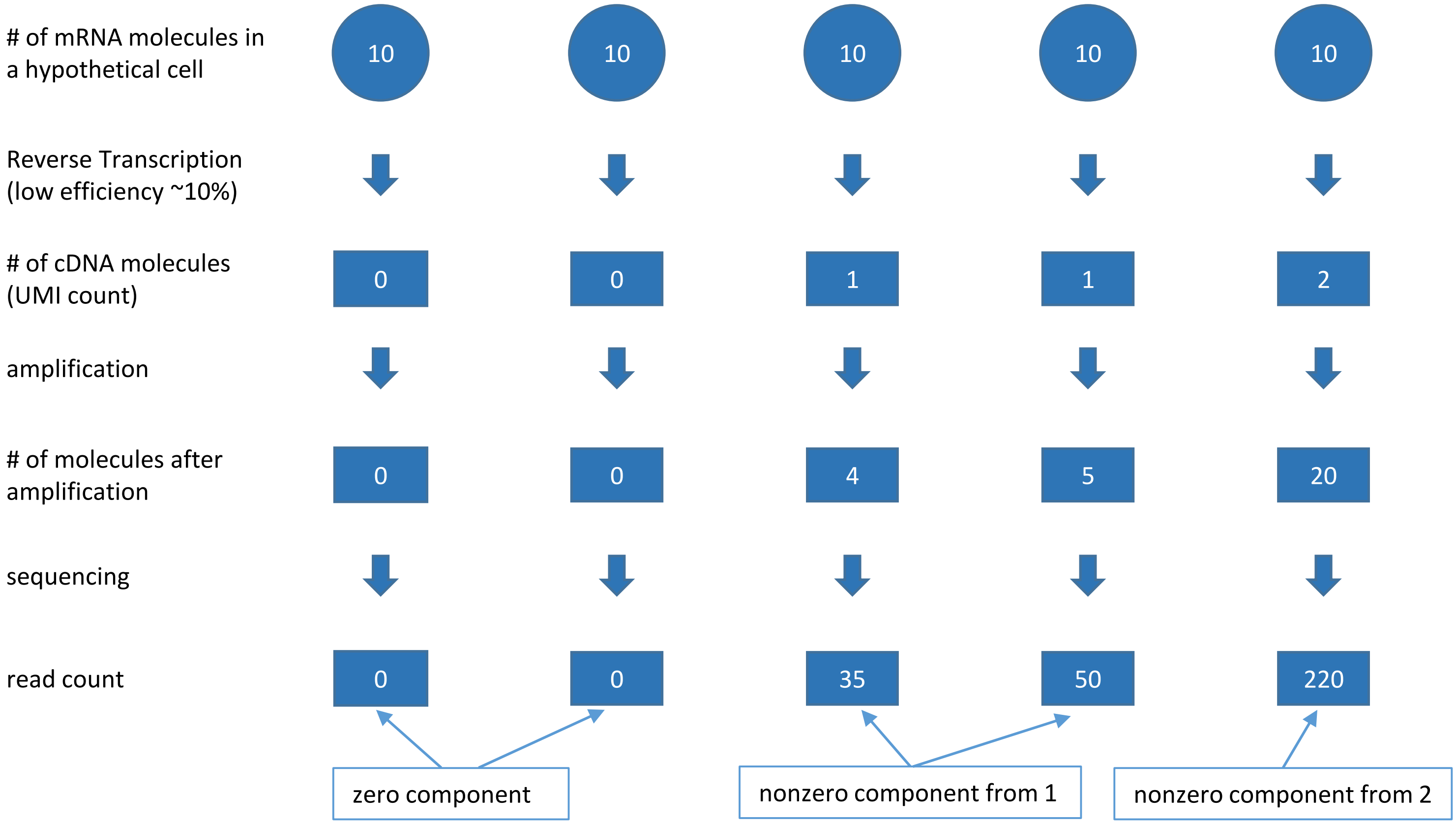

Figure S12. A model illustrating the generation of UMI counts and read counts. The counts in the figure are hypothetical for illustrating the effects of each processing step.
